# A novel staging system of cardiac damage in aortic stenosis based on multi-chamber myocardial deformation

**DOI:** 10.1093/ehjci/jeaf035

**Published:** 2025-01-28

**Authors:** Michele Tomaselli, Paolo Springhetti, Giovanni Benfari, Marco Penso, Alexandra Clement, Matteo Pilan, Denis Leonardi, Luca Ciceri, Alexandra Buta, Roberto Scarsini, Andreea Calin, Claudia Nitu, Noela Radu, Denisa Muraru, Bogdan A Popescu, Flavio Ribichini, Luigi P Badano

**Affiliations:** Department of Cardiology, Istituto Auxologico Italiano, IRCCS, Milan, Italy; Department of Medicine, Division of Cardiology, University of Verona, Piazzale Aristide Stefani 1, 37100 Verona, Italy; Department of Medicine, Division of Cardiology, University of Verona, Piazzale Aristide Stefani 1, 37100 Verona, Italy; Department of Cardiology, Istituto Auxologico Italiano, IRCCS, Milan, Italy; Internal Medicine Department, ‘Grigore T. Popa’, University of Medicine and Pharmacy, Iasi, Romania; Department of Medicine, Division of Cardiology, University of Verona, Piazzale Aristide Stefani 1, 37100 Verona, Italy; Department of Medicine, Division of Cardiology, University of Verona, Piazzale Aristide Stefani 1, 37100 Verona, Italy; Department of Medicine, Division of Cardiology, University of Verona, Piazzale Aristide Stefani 1, 37100 Verona, Italy; Cardiology Department, University of Medicine and Pharmacy ‘Carol Davila’, Emergency Institute for Cardiovascular Diseases ‘Prof. Dr. C. C. Iliescu’, Bucharest, Romania; Department of Medicine, Division of Cardiology, University of Verona, Piazzale Aristide Stefani 1, 37100 Verona, Italy; Cardiology Department, University of Medicine and Pharmacy ‘Carol Davila’, Emergency Institute for Cardiovascular Diseases ‘Prof. Dr. C. C. Iliescu’, Bucharest, Romania; Cardiology Department, University of Medicine and Pharmacy ‘Carol Davila’, Emergency Institute for Cardiovascular Diseases ‘Prof. Dr. C. C. Iliescu’, Bucharest, Romania; Department of Cardiology, Istituto Auxologico Italiano, IRCCS, Milan, Italy; Department of Cardiology, Istituto Auxologico Italiano, IRCCS, Milan, Italy; Department of Medicine and Surgery, University of Milano-Bicocca, Milan, Italy; Cardiology Department, University of Medicine and Pharmacy ‘Carol Davila’, Emergency Institute for Cardiovascular Diseases ‘Prof. Dr. C. C. Iliescu’, Bucharest, Romania; Department of Medicine, Division of Cardiology, University of Verona, Piazzale Aristide Stefani 1, 37100 Verona, Italy; Department of Cardiology, Istituto Auxologico Italiano, IRCCS, Milan, Italy; Department of Medicine and Surgery, University of Milano-Bicocca, Milan, Italy

**Keywords:** aortic stenosis, speckle tracking echocardiography, outcome, cardiac damage

## Abstract

**Aims:**

This study evaluates whether multi-chamber myocardial deformation analysis using speckle tracking echocardiography (STE) can enhance validated current staging systems and improve risk stratification for patients with moderate-to-severe aortic stenosis (AS).

**Methods and results:**

We reanalysed 2D, Doppler, and STE data obtained from two cohorts: derivation (654 patients, median age: 82 years; 51% men) and validation (237 patients, median age: 77 years; 55% men) with at least moderate AS (aortic valve area < 1.5 cm^2^). The receiver operator characteristic curve analysis identified optimal cut-off values linked to outcomes: 15% for left ventricular global longitudinal strain (LVGLS), 13% for peak atrial longitudinal strain (PALS), and 19% for right ventricular free-wall strain (RVFWS). Patients have been divided into five stages: Stage 0, no left-side damage (LVGLS ≥ 15% and PALS ≥ 13%); Stage 1, partial left-side damage (LVGLS < 15% or PALS < 13%); Stage 2, definite left-side damage (LVGLS < 15% and PALS < 13%); Stage 3, no right-side damage (RVFWS ≥ 19%); and Stage 4, right-side damage (RVFWS < 19%). In a multivariable Cox regression analysis, the new staging scheme remained independently associated with an increased risk of all-cause death [adjusted-hazard ratio: 1.28; 95% confidence interval (CI): 1.10–1.48; *P* = 0.001]. This new staging classification exhibited higher predictive power [area under the curve (AUC) 0.67; 95% CI 0.62–0.73] than those proposed by Généreux *et al*. (Staging classification of aortic stenosis based on the extent of cardiac damage. Eur Heart J 2017;38:3351–8.) (AUC 0.62; 95% CI 0.56–0.67; *P* = 0.002) and Tastet *et al*. (Staging cardiac damage in patients with asymptomatic aortic valve stenosis. J Am Coll Cardiol 2019;74:550–63.) (AUC 0.64; 95% CI 0.58–0.70; *P* = 0.041) for 2-year all-cause death, with similar findings in the validation cohort.

**Conclusion:**

Our staging system for detecting cardiac damage, incorporating multi-chamber myocardial deformation, exhibits a stronger association with outcomes than previously validated systems.

## Graphical Abstract

**Graphical Abstract:**
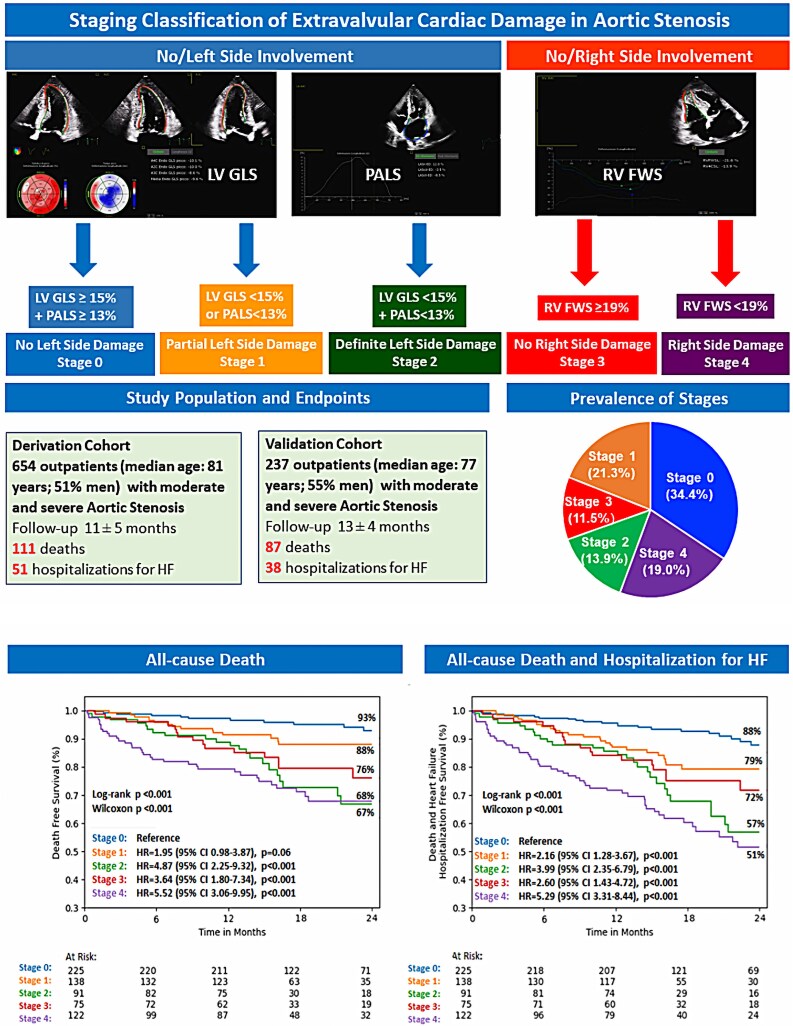
Criteria for defining cardiac damage stages using multi-chamber myocardial deformation parameters, along with characteristics and prevalence in the derivation cohort, and their association with outcomes. CI, confidence interval; HR, hazard ratio; LVGLS, left ventricular longitudinal strain; PALS, peak atrial longitudinal strain; RVFWS, right ventricular free-wall longitudinal strain.

## Introduction

Aortic stenosis (AS) is a burdensome health problem with an incidence expected to increase in the coming years due to the aging of the population.^1^ Although surgical aortic valve replacement (SAVR) and transcatheter aortic valve replacement (TAVR) are increasingly being performed in symptomatic patients with severe AS,^2^ the mortality associated with the disease remains still significant.^3,4^ Emerging evidence suggests that in patients with severe AS undergoing TAVR, assessing extravalvular cardiac damage may provide useful prognostic insights^5^ and identify specific features for early interventions.^6^ Nevertheless, this framework predominantly relies on conventional echocardiography, which has limited sensitivity in detecting subclinical myocardial damage resulting from prolonged AS-related afterload.^7–9^ A refined staging system incorporating left ventricular (LV) global longitudinal strain (GLS) provides enhanced prognostic insights over the original system in asymptomatic moderate-to-severe AS patients.^10^ Moreover, preliminary data showed the incremental prognostic value of assessing the myocardial deformation of the LV,^7^ the left atrium (LA),^9^ or the right ventricle (RV)^8,11^ by 2D speckle tracking echocardiography (2D-STE) in AS. However, the clinical impact of evaluating extravalvular cardiac damage by incorporating multi-chamber myocardial deformation has never been tested. Therefore, our goal was to develop a novel staging system for extravalvular cardiac damage using 2D-STE-derived myocardial deformation parameters from the LV, LA, and RV and assess its association with outcomes in moderate-to-severe AS patients.

## Methods

### Study population

Two separate cohorts were used to develop and validate our novel staging system of extravalvular cardiac damage in patients with AS. The derivation cohort included outpatients with moderate and severe AS who underwent transthoracic echocardiograms at two medical centres: Istituto Auxologico Italiano, San Luca Hospital, in Milan, and the University Hospital of Verona, Verona, between January 2019 and June 2022. The external validation cohort included patients with moderate-to-severe AS who underwent transthoracic echocardiograms prior to TAVR/SAVR at the Emergency Institute for Cardiovascular Diseases Prof. Dr. C.C. Iliescu in Bucharest, Romania, between January 2018 and February 2021. The study inclusion criteria were at least moderate AS [peak aortic jet velocity ≥ 3 m/s or transaortic mean gradient ≥ 20 mmHg or aortic valve area (AVA) ≤ 1.5 cm^2^].^12^ Exclusion criteria were poor image quality, greater than or equal to moderate mitral stenosis and/or aortic regurgitation, previous cardiac surgery/transcatheter interventions, cardiac amyloidosis, hypertrophic obstructive cardiomyopathy, and missing relevant clinical data. The study was conducted in accordance with the respective institutional guidelines and the Helsinki Declaration.

### Echocardiography

Patients underwent thorough 2D and Doppler echocardiography, which was quantitated offline using Image Arena 4.6 (TOMTEC Imaging Systems, Munich, Germany). Two blinded experienced researchers (P.S. and A.C.) conducted the quantitative analyses according to ASE/EACVI guidelines on cardiac chamber quantitation.^13^ LVGLS, peak LA longitudinal strain (PALS), and RV free-wall strain (RVFWS) were measured using current recommendations^14,15^ and reported in absolute values. In patients with atrial fibrillation (AF), LVGLS was measured, when possible, using a triplanar acquisition method, allowing simultaneous capture of all three apical views in a single cardiac cycle by selecting an RR interval that corresponds to a heart rate between 60 and 90  bpm. The same RR interval selection principle was applied to measure PALS and RVFWS. AS severity was assessed by measuring the maximum aortic velocity using continuous-wave Doppler with systematic multiple-window interrogation.^12,16^ The peak transaortic pressure gradient was computed using the simplified Bernoulli’s equation, while the mean pressure gradient was averaged over the ejection period.^12,16^ The LV outflow tract diameter was measured from a zoomed parasternal long-axis view in mid-systole parallel to the aortic valve (AV) plane.^12,16^ The stroke volume and the AVA were calculated using the continuity equation.^12,16^ The classification of AS severity followed the current recommendations.^12,16–18^ All the measurements were obtained by averaging three consecutive cardiac cycles (five for patients with AF).

### Proposed staging system

The echocardiographic parameters included in the cardiac damage staging classification proposed by Généreux *et al*.^5^ and Tastet *et al*.^10^ are presented in the Supplementary data online, *Table S1*. To develop the new staging system of extravalvular cardiac damage, we initially identified patients with signs of right-side damage (Group 1), following Généreux *et al*. definition.^5^ These criteria included a pulmonary artery systolic pressure (PASP) > 60 mmHg, moderate-to-severe TR or RV systolic dysfunction, which is defined as RV S′ < 9.5 cm/s, and/or tricuspid annular plane systolic excursion (TAPSE) < 17 mm. Based on RVFWS values, Group 1 patients were subdivided into two categories: Stage 3, with normal RVFWS (≥19%), and Stage 4, with abnormal RVFWS (<19%). The remaining population (Group 0) was divided into three stages based on the extent of left-side damage using LVGLS and PALS: Stage 0, no left-side damage, characterized by LVGLS ≥ 15% and PALS ≥ 13%; Stage 1, partial left-side damage, presenting either reduced LVGLS (<15%) or PALS (<13%); and Stage 2, definite left-side damage, presenting both LVGLS (<15%) and PALS (<13%) impairment.

### Follow-up and study endpoint

The primary endpoint was all-cause mortality, while the secondary endpoint was a composite of all-cause mortality and/or hospitalization for heart failure (HF). Moreover, we also examined outcomes under medical management, by censoring patients at the time of AVR. Patients were followed until they either experienced an event, underwent AVR, or had their last medical contact. Survival data and hospitalization were gathered regularly through telephone interviews, consultation with the patient’s physicians, and review of electronic medical records. Mortality status was independently verified through death certificates. Physicians, blinded to echocardiographic/clinical data, assigned clinical events.

### Statistical analysis

Statistical analyses were performed using IBM-SPSS Statistic, v28.0 and R-v4.3.1. A two-sided significance level of *P* < 0.05 was considered statistically significant. The normal distribution of continuous variables was tested with the Shapiro–Wilk test. Continuous data were expressed as mean ± SD or median [interquartile range (IQR)]. Comparison of continuous data was performed using Student’s *t*-test or Wilcoxon–Mann–Whitney test as appropriate. ANOVA test and the *post hoc* Bonferroni correction assessed group differences. Categorical variables are reported as counts and percentages and were compared using the *χ*² or Fisher’s exact tests, as appropriate. Receiver operator characteristic (ROC) curves were created to determine the optimal cut-off values of LVGLS, PALS, and RVFWS in predicting outcomes, using Youden’s index. The resulting cut-offs were 15% for LVGLS, 13% for PALS, and 19% for RVFWS. Reproducibility of LVGLS, PALS, and RVFWS measurements was assessed using intraclass correlation coefficients (ICCs) and coefficients of variation to evaluate interobserver and intraobserver variability. Kaplan–Meier curves were employed to estimate event-free survival rates, and group differences were analysed using both the log-rank test and the Wilcoxon test. A multivariable Cox proportional hazards model was used to determine the association between the proposed staging classification and the outcome. The model was adjusted for age, sex, previous myocardial infarction, AF, NYHA class ≥ III, diabetes, renal disease, AS severity, and AVR. Variables for multivariable analysis were selected based on clinical relevance and/or significant association with mortality and HF hospitalization in the univariable analysis. Results are presented as hazard ratio (HR) with the corresponding 95% confidence interval (CI). ROC curve analysis, net reclassification improvement, and integrated discrimination improvement were used to evaluate the proposed staging classification’s ability to predict 2-year risk of endpoints. The incremental value of the proposed staging scheme over conventional echocardiographic variables was evaluated by comparing the increase in *χ*² value of combined hierarchical models.

## Results

### Derivation cohort—characteristics

A total of 1045 patients with moderate-to-severe AS underwent screening. Of these, 654 patients were included in the final analysis (see Supplementary data online, *Figure S1*). The characteristics of the study cohorts are summarized in Supplementary data online, *Table S2*. Calcific-degenerative AS was the predominant aetiology (589 out of 654, 90%), while the remaining cases involved patients with congenital-bicuspid AV (9%) and rheumatic AS (1%). Low-flow low-gradient AS was present in 24% of patients. Most of the patients were paucisymptomatic (NYHA I–II, 77%). The patients’ distribution according to the new cardiac damage staging scheme (*Graphical Abstract*) was significantly different compared with the previously published ones (see Supplementary data online, *Figure S2*). Our patients in the most advanced stages of cardiac damage (i.e. stage ≥ 3) were older and more symptomatic, had a higher prevalence of significant chronic kidney disease, more previous ischaemic heart disease, more hospitalizations for HF, and a higher incidence of moderate-to-severe MR and TR (*P* < 0.05, for all). Moreover, our patients in stages ≥ 3 presented larger LV dimensions, a higher incidence of LV systolic and diastolic dysfunction, higher PASP, and more impaired LVGLS, PALS, and RVFWS (*P* < 0.001) (*Table 1*). The characteristics of the study population according to the previously reported staging systems are summarized in the Supplementary data online, *Tables S3* and *S4*.

**Table 1:** Characteristics of the study population

	Stage 0 (*n* = 225)	Stage 1 (*n* = 139)	Stage 2 (*n* = 91)	Stage 3 (*n* = 75)	Stage 4 (*n* = 124)	*P* value
Demographics						
Age (years)	80 (79–81)	81 (79–82)	82 (81–84)^†^	84 (83–86)^†,‡^	83 (82–85)^†,‡^	**<0**.**001**
Male	98(44)	78(56)	56(62)	35(47)	69(56)	**0**.**017**
Body surface area (m^2^)	1.78 (1.75–1.80)	1.78 (1.74–1.82)	1.82 (1.78–1.87)	1.73 (1.68–1.77)	1.78 (1.74–1.82)	0.074
Body mass index (kg/m^2^)	26.5 (25.8–27.1)	26.3 (25.5–27.1)	26.0 (24.9–27.0)	25.7 (24.6–26.8)	25.7 (24.9–26.5)	0.736
Smoking	51 (23)	41 (30)	27 (30)	11 (15)	25 (20)	0.068
Hypertension	134 (60)	85 (61)	61 (67)	55 (73)	63 (51)	**0**.**019**
Diabetes	54 (24)	44 (32)	34 (37)	14 (19)	30 (24)	**0**.**034**
Dyslipidaemia	148 (66)	71 (52)	43 (47)	37 (51)	71 (57)	**0**.**008**
NYHA ≥ III	38 (17)	49 (36)	39 (43)	23 (32)	70 (57)	**<0**.**001**
Angina	45 (20)	26 (19)	15 (17)	16 (22)	13 (11)	0.167
Syncope	15 (7)	16 (12)	10 (11)	7 (10)	11 (9)	0.554
COPD	15 (7)	13 (9)	7 (8)	10 (13)	12 (10)	0.464
Coronary artery disease	62 (28)	51 (37)	28 (31)	26 (35)	50 (40)	0.129
Previous myocardial infarction	26(12)	25 (18)	17 (19)	13 (18)	41 (33)	**<0**.**001**
Previous acute heart failure	14 (6)	29 (21)	19 (21)	16 (22)	51 (42)	**<0**.**001**
eGFR (mL/min/m^2^)	62 (59–65)	55 (51–59)	52 (48–56)	49 (44–55)	48 (44–52)	**<0**.**001**
Systolic blood pressure (mmHg)	139 (137–142)	140 (137–144)	139 (134–143)	138 (133–142)	127 (124–131)	**<0**.**001**
Diastolic blood pressure (mmHg)	75 (74–77)	76 (74–79)	79 (76–82)	75 (72–78)	74 (72–75)	**0**.**036**
Atrial fibrillation at TTE	8 (4)	13 (9)	36 (40)	24 (32)	62 (50)	**<0**.**001**
Aortic stenosis						**0**.**004**
Moderate	99 (44)	55 (40)	24 (26)	39 (52)	41 (33)	
Severe	126 (56)	84 (60)	67 (74)	36 (48)	83 (70)	
TTE characteristics						
Relative wall thickness	0.55 (0.49–0.61)	0.53 (0.47–0.58)	0.55 (0.45–0.65)	0.49 (0.47–0.52)	0.49 (0.46–0.52)	0.065
Septal wall thickness, (cm)	1.3 (1.2–1.3)	1.3 (1.2–1.3)	1.3 (1.3–1.4)	1.2 (1.2–1.3)	1.3 (1.2–1.3)	0.117
Posterior wall thickness (cm)	1.1 (1.1–1.1)	1.1 (1.1–1.2)	1.1 (1.1–1.2)	1.0 (1.0–1.1)	1.2 (1.1–1.3)	0.068
LV end-diastolic diameter (cm)	4.4 (4.3–4.4)	4.6 (4.5–4.7)^†^	4.7 (4.5–4.9)^†^	4.6 (4.4–4.7)^†^	5.0 (4.6–5.3)^†,‡,||^	**<0**.**001**
LV mass (g/m^2^)	103 (99–107)	119 (114–125)^†^	121 (114–128)^†^	111 (103–118)^‡,§^	126 (119–134)^†,||^	**<0**.**001**
LV end-diastolic volume (mL/m^2^)	59 (57–60)	65 (62–69)^†^	64 (60–69)	58 (53–63)^‡,§^	69 (64–73)†||	**<0**.**001**
LV end-systolic volume (mL/m^2^)	22 (21–22)	29 (27–32)^†^	32 (29–36)^†^	25 (21–29)^‡,§^	39 (35–43)^†,‡,||^	**<0**.**001**
LV stroke volume (mL/m^2^)	42 (41–43)	38 (37–39)^†^	33 (31–34)^†,‡^	36 (34–37)^†,‡,§^	30 (29–32)^†,‡,§,||^	**<0**.**001**
LV ejection fraction (%	)63.7 (63.0–64.4)	57.2 (55.4–59.0)^†^	52.3 (49.8–54.8)^†,‡^	58.9 (56.3–61.4)^†,§^	46.7 (44.1–49.4)^†,‡,§,||^	**<0**.**001**
Left atrial volume (mL/m^2^)	42 (41–43)	45 (43–47)^†^	55 (52–59)^†,‡^	57 (52–62)^†,‡^	61 (58–64)^†,‡,§,||^	**<0**.**001**
E/e′ ratio	12.4 (11.9–13.0)	14.0 (13.1–14.8)^†^	15.0 (14.0–16.0)^†^	14.7 (13.6–15.9)^†^	16.4 (15.5–17.3)^†,‡,§,||^	**<0**.**001**
MR ≥ moderate	25 (11)	27 (19)	21 (23)	41 (55)	74 (60)	**<0**.**001**
PASP (mmHg)	33 (32–34)	35 (33–36)^†^	39 (37–41)^†,‡^	49 (46–52)^†,‡,§^	50 (47–53)^†,‡,§^	**<0**.**001**
TR ≥ moderate	0 (0)	0 (0)	0 (0)	56 (75)	84 (68)	**<0**.**001**
Mean aortic valve gradient (mmHg)	34 (32–36)	34 (31–38)	34 (30–37)	27 (24–31)^†,‡,§^	25 (23–28)^†,‡,§^	**<0**.**001**
Aortic valve area (cm^2^)	0.90 (0.86–0.93)	0.85 (0.81–0.90)	0.77 (0.71–0.83)^†,‡^	0.88 (0.82–0.95)^§^	0.85 (0.80–0.91)^†,§^	**0**.**002**
LV global LS (%)	18.1 (17.8–18.4)	13.2 (12.8–13.7)^†^	10.5 (9.9–11.0)^†,‡^	14.4 (13.5–15.3)^†,‡,§^	9.9 (9.1–10.6)^†,‡,||^	**<0**.**001**
RV free-wall LS (%)	24.0 (23.4–24.7)	22.7 (21.9–23.6)^†^	19.2 (18.0–20.4)^†,‡^	23.5 (22.6–24.4)^§^	12.8 (12.1–13.5)^†,‡,§,||^	**<0**.**001**
Peak atrial LS (%)	25.7 (24.8–26.7)	19.6 (18.4–20.7)^†^	9.3 (8.7–9.8)^†,‡^	14.4 (12.7–16.0)^†,‡,§^	9.0 (8.0–10.0)^†,‡,§,||^	**<0**.**001**

Values: mean (95% CI) or *n* (%). *P* values show differences between stages of extra-aortic valvular cardiac: ^†^*P* < 0.05 vs. Group 0; ^‡^*P* < 0.05 vs. Group 1; ^§^*P* < 0.05 vs. Group 2; ^||^*P* < 0.05 vs. Group 3. Bold values represent *P* < 0.05.

COPD, chronic obstructive pulmonary disease; GFR, glomerular filtration rate; GLS, global longitudinal strain; LV, left ventricle; LS, longitudinal strain; MR, mitral regurgitation; PASP, pulmonary artery systolic pressure; RV, right ventricle; TR, tricuspid regurgitation.

### Survival analysis

During a median follow-up of 11 (IQR: 5–17) months, patients experienced 111 deaths and 51 hospitalizations for HF. Three-hundred and twenty-five (50%) patients were referred for AVR (303 TAVR and 22 SAVR) at a median time of 221 (IQR: 3–597) days during follow-up. As expected, the patients who did not undergo AVR experienced a higher incidence of death (25%, *n* = 83, median time 260 [IQR: 105–486] days), compared with those who underwent AVR (9%, *n* = 28, with a median time of 371 [IQR: 201–597] days) (see Supplementary data online, *Table S5*).

**Figure 1:**
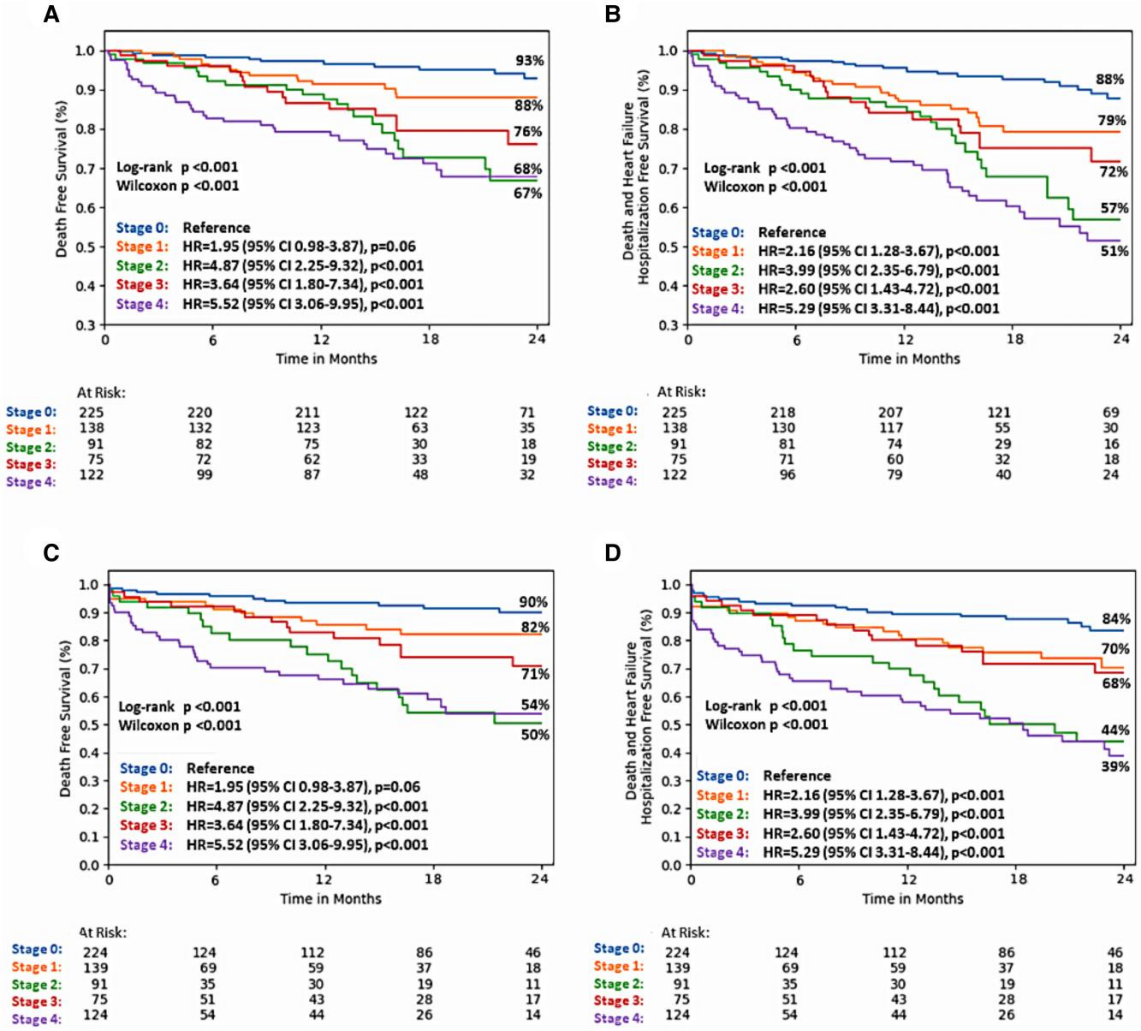
The Kaplan–Meier analyses for primary (*A*) and secondary (*B*) endpoints. Similar analyses censored at the time of aortic valve replacement for primary (*C*) and secondary (*D*) endpoints.

*Table 2* shows the parameters associated with the primary and the secondary endpoints for univariable Cox regression analysis. The Kaplan–Meier analysis according to the new staging scheme revealed a significantly higher incidence of cardiovascular events for each stage of cardiac damage compared with Stage 0 at 2-year follow-up (*P* < 0.001) (*Figure 1*). Specifically, patients in Stage 2 demonstrated a lower survival rate compared with those in Stage 3 at 2 years (54% vs. 71% for all-cause death and 44% vs. 68% for the composite outcome). These findings were confirmed even after censoring at the time of AVR (*Figure 1*). The Kaplan–Meier curves based on the Généreux *et al*.^5^ and Tastet *et al*.^10^ staging schemes are presented in Supplementary data online, *Figures S3* and *S4*. The probability of mortality for each stage was 6.6% for Stage 0, 10.3% for Stage 1, 22.8% for Stage 2, 20.6% for Stage 3, and 26.7% for Stage 4(see Supplementary data online, *Figure S5A*). For the combined endpoint, the probability for each stage was 10.2% for Stage 0, 17.2% for Stage 1, 29.3% for Stage 2, 26.6% for Stage 3, and 39.6% for Stage 4 (see Supplementary data online, *Figure S5B*).

**Table 2:** Association of the proposed staging scheme with outcomes

	Univariable		Multivariable	
	HR (95% CI)	*P* value	HR (95% CI)	*P* value
All-cause mortality				
Age	1.10 (1.06–1.13)	**<0**.**001**	1.03 (0.99–1.07)	0.136
Male	1.04 (0.71–1.51)	0.837	1.209 (0.80–1.82)	0.364
Previous myocardial infarction	1.89 (1.26–2.83)	**0**.**002**	1.26 (0.63–2.52)	0.511
Atrial fibrillation	2.03 (1.37–3.02)	**<0**.**001**	1.11 (0.72–1.73)	0.613
NYHA ≥ III	1.80 (1.23–2.63)	**0**.**002**	1.19 (0.78–1.82)	0.408
eGFR	0.96 (0.95–0.97)	**<0**.**001**	0.985 (0.97–0.99)	**0**.**018**
Diabetes	0.95 (0.62–1.46)	0.841	1.14 (0.73–1.79)	0.549
Aortic valve area	0.56 (0.28–1.12)	0.102	0.49 (0.19–1.26)	0.139
Surgical/transcatheter AVR	0.34 (0.22–0.53)	**<0**.**001**	0.32 (0.18–0.56)	**<0**.**001**
Severe AS	1.08 (0.73–1.58)	0.685	1.65 (0.95–2.85)	0.072
Stage of cardiac damage (each stage increase)	1.62 (1.35–1.94)	**<0**.**001**	1.28 (1.10–1.48)	**<0**.**001**
Stage 0 (reference)				
Stage 1			1.65 (0.82–3.32)	**0**.**163**
Stage 2			2.99 (1.49–5.99)	**0**.**002**
Stage 3			2.04 (0.98–4.26)	**0**.**045**
Stage 4			3.11 (1.57–6.15)	**0**.**001**
Death and/or HF				
Age	1.07 (1.04–1.10)	**<0**.**001**	1.02 (0.09–1.05)	0.184
Male	0.97 (0.71–1.32)	0.863	1.11 (0.79–1.56)	0.536
Previous myocardial infarction	1.53 (1.08–2.18)	**0**.**016**	1.10 (0.62–1.93)	0.741
Atrial fibrillation	2.34 (1.69–3.23)	**<0**.**001**	1.45 (1.01–2.07)	**0**.**041**
NYHA ≥ III	2.06 (1.51–2.82)	**<0**.**001**	1.51 (1.07–2.12)	**0**.**018**
eGFR	0.97 (0.97–0.98)	**<0**.**001**	0.99 (0.98–1.00)	0.103
Diabetes	1.04 (0.74–1.47)	0.806	1.18 (0.83–1.70)	0.334
Aortic valve area	0.44 (0.25–0.77)	**0**.**005**	0.55 (0.25–1.20)	0.135
Surgical/transcatheter AVR	0.62 (0.45–0.86)	**0**.**004**	0.62 (0.39–0.97)	**0**.**037**
Severe AS	1.27 (0.93–1.75)	0.130	1.40 (0.87–2.26)	0.156
Stage of cardiac damage (each stage increase)	1.42 (1.29–1.57)	**<0**.**001**	1.28 (1.13–1.45)	**<0**.**001**
Stage 0 (reference)				
Stage 1			1.76 (1.02–3.01)	**0**.**041**
Stage 2			2.30 (1.29–4.09)	**0**.**005**
Stage 3			1.63 (0.87–3.05)	0.125
Stage 4			3.25 (1.88–5.62)	**<0**.**001**

Bold values represent *P* < 0.05.

After conducting a Cox regression analysis, we found that our staging scheme remained independently associated with an increased risk of both primary [adjusted HR (aHR): 1.28; 95% CI: 1.10–1.48; *P* = 0.001] and secondary endpoints (aHR: 1.28; 95% CI: 1.13–1.45; *P* < 0.001) (*Table 2*).

### Incremental prognostic value of the proposed staging scheme

**Figure 2:**
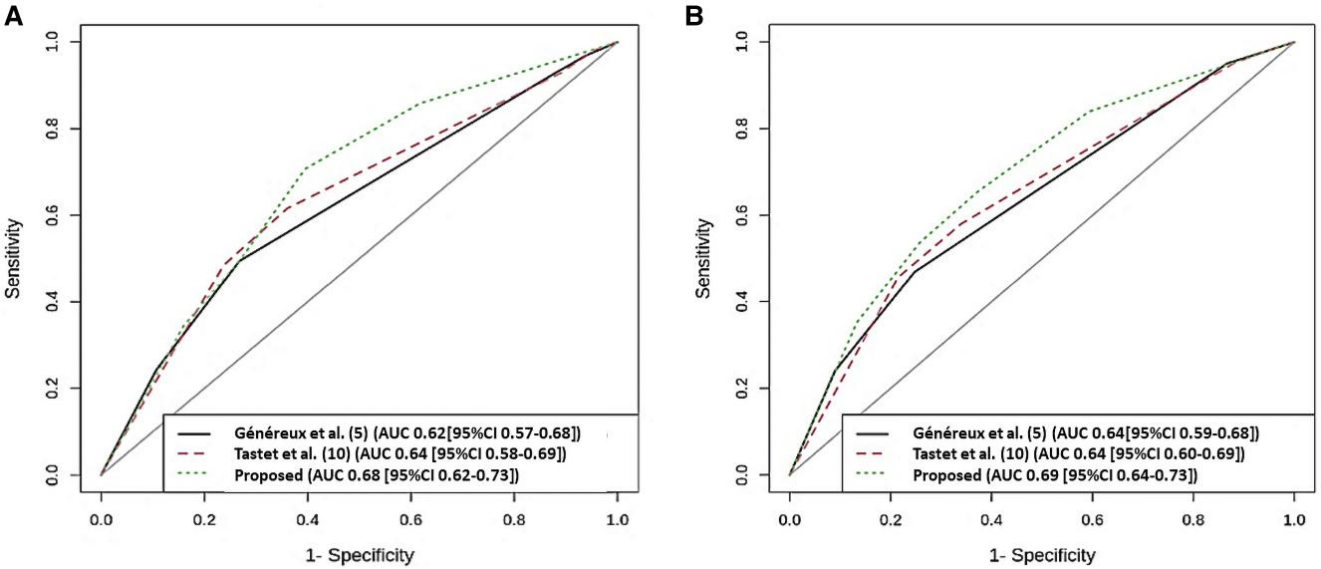
Time-dependent ROC curves comparing the proposed and previously reported staging models for the primary (*A*) and secondary (*B*) endpoints at 2 years. AUC, area under the curve.

According to the ROC curve analysis (*Figure 2*), the proposed staging classification showed a significantly closer association with the primary endpoint [0.67 (95% CI 0.62–0.73)] compared with the classifications proposed by Généreux [0.62 (95% CI 0.56–0.67); *P* = 0.002] and Tastet [0.64 (95% CI 0.58–0.70); *P* = 0.041] at 2 years. Moreover, our staging classification displayed significantly closer association with the secondary outcome [0.69 (95% CI 0.64–0.73)] compared with Généreux’s [0.64 (95% CI 0.59–0.68); *P* = 0.014] and Tastet’s [0.64 (95% CI 0.60–0.60); *P* = 0.047] classifications at 2 years. Similar results are obtained with the reclassification analysis at 2 years (see Supplementary data online, *Table S6*). Finally, the proposed staging system showed significant incremental prognostic value over conventional echocardiography for both primary (*P* < 0.001) and secondary outcomes (*P* = 0.023) at hierarchical model *χ*² analysis (see Supplementary data online, *Figure S6*).

### Staging classification according to AS severity

**Figure 3:**
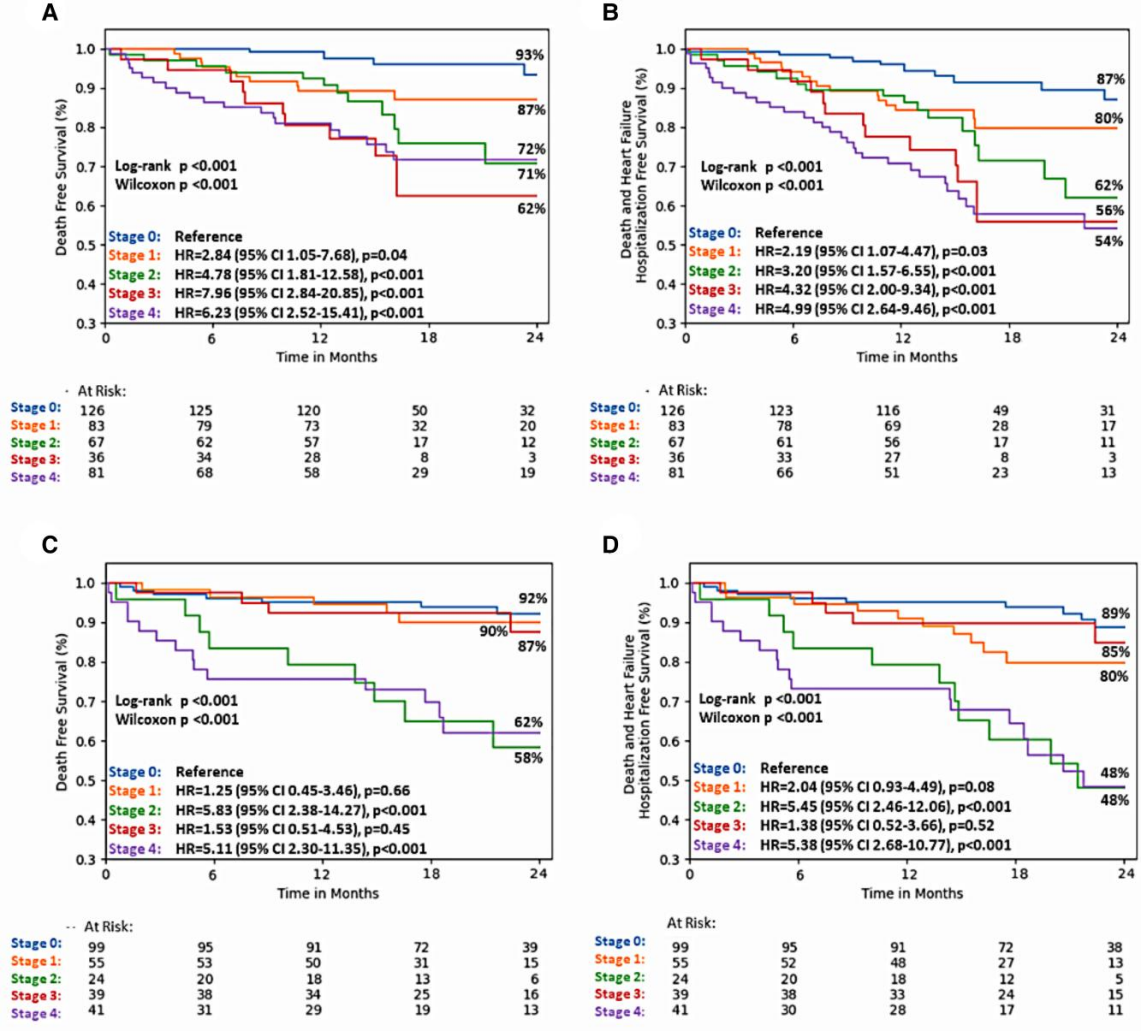
The Kaplan–Meier analyses for primary and secondary endpoints in patients with severe (*A* and *B*) and moderate aortic stenosis (*C* and *D*). Abbreviations in *Figures 1* and *3* as in *Graphical Abstract*.

According to the new staging system, 31.8% of the 396 patients with severe AS were in Stage 0, 21.2% in Stage 1, 16.9% in Stage 2, 9.1% in Stage 3, and 21.0% in Stage 4. The Kaplan–Meier analysis revealed that stages characterized by RV involvement in this group of patients were at a higher risk of experiencing adverse events (*Figure 3A* and *B*). In multivariable analysis, the new cardiac damage staging was significantly associated with all-cause mortality (*P* < 0.001) and the combined endpoint (*P* < 0.001) in patients with severe AS (see Supplementary data online, *Table S7*).

According to the new staging scheme, 38.4% of the 258 patients with moderate AS were classified as Stage 0, 21.3% as Stage 1, 9.3% as Stage 2, 15.1% as Stage 3, and 15.9% as Stage 4. The Kaplan–Meier plots (*Figure 3C* and *D*) in these patients revealed that the groups in Stages 2 and 4 experienced the lowest survival rates for primary (62% and 58%, respectively) and secondary endpoint (48%, both groups). Specifically, patients in Groups 2 and 4 had a five-fold higher risk of experiencing cardiovascular events compared with those in Group 0. In multivariable analysis, the proposed staging was independently associated only with the combined endpoint (*P* = 0.045) (see Supplementary data online, *Table S8*).

### External validation

As expected, patients in the validation cohort were younger and sicker than those in the derivation cohort (see Supplementary data online, *Table S2*). The validation cohort was divided into five stages based on the proposed staging system (see Supplementary data online, *Table S9*). Over a median 13-month follow-up (IQR: 5–20), there were 87 deaths and 38 HF hospitalizations. The Kaplan–Meier analysis showed the highest 2-year event rates in Stages 2 and 4 (*P* < 0.001) (see Supplementary data online, *Figure S7A* and *B*). Cox regression results for primary and secondary endpoints are presented in Supplementary data online, *Table S10*. The proposed staging scheme showed a stronger correlation with both endpoints than Généreux’s^5^ and Tastet’s^10^ at 2 years (*P* < 0.001), with added predictive value in the reclassification analysis (see Supplementary data online, *Table S11 and Figure S6C* and *D*).

### Reproducibility of strain measurements

ICC for intra- and interobserver reproducibility of longitudinal strain measurements ranged from 0.95 to 0.98 (see Supplementary data online, *Table S12*).

## Discussion

This study is the first to assess the prognostic value of a staging system for extravalvular cardiac damage using multi-chamber myocardial deformation in patients with moderate-to-severe AS. The 2D-STE staging system offered additional predictive insights beyond traditional echocardiographic measures and previous classifications^5,10^ in predicting medium-term all-cause mortality and HF hospitalizations.

### The use of 2D-STE provides a valuable tool for evaluating cardiac damage in AS

Assessing AS severity and patients’ functional capacity is crucial to determine the timing for AVR and to predict patients’ prognosis.^17,18^ However, this approach, often limited by discordant results regarding disease severity,^3,12^ is oversimplified, as clinical outcomes are mainly driven by cardiac dysfunction.^5,6^ To address this complexity, Généreux *et al*.^5^ proposed a cardiac staging model, which was tested in 1661 patients with symptomatic severe AS undergoing SAVR or TAVR. For each stage increment, the 1-year mortality risk increased by approximately 45%. While this conceptual framework^5^ provides an elegant physiological progression of myocardial involvement in patients with significant AS, it is limited by conventional echocardiographic parameters, which are less sensitive in detecting subclinical myocardial damage from AS.^7^

Recent studies indicate that LVGLS is a better survival predictor than LV ejection fraction in asymptomatic patients with moderate-to-severe AS and preserved LV function^19,20^ and even after TAVR.^21^ A meta-analysis identified an optimal LVGLS threshold of 14.7% for high-risk patients,^20^ closely matching our analysis.

A study of 735 asymptomatic patients with moderate-to-severe AS and LV ejection fraction > 50% incorporated LVGLS into a refined AS staging system, providing better prognostic information than the conventional staging system,^10^ confirmed by further evidence.^22^

Growing evidence suggests that PALS is a better predictor of prognosis in moderate-to-severe AS patients than LV ejection fraction or LVGLS.^9^ Moreover, PALS has a complementary roles on LA volume (LAV) in assessing atrial pathophysiology in AS, with LAV reflecting long-term atrial remodelling due to chronic pressure or volume overload and PALS capturing early functional impairments associated with subclinical changes in atrial mechanics.^9^ We found a 13% PALS cut-off suggesting the highest risk for adverse cardiovascular events, lower than previously reported.^9^ Furthermore, RV dysfunction is the most significant predictor of mortality in patients with AS, and it was prevalent in many patients with severe AS undergoing AVR.^23^ Additionally, RVFWS demonstrated incremental prognostic value for all-cause mortality at 1 year in high-risk AS patients undergoing TAVR.^8^ Limited data exist on multi-chamber longitudinal strain in AS. In a retrospective analysis of patients with at least moderate AS, applying LVGLS, RVFWS, and PALS simultaneously showed a strong association with all-cause mortality.^24^

### Clinical utility of the cardiac damage staging system

Our staging system included a broad range of moderate and severe AS patients, both symptomatic and asymptomatic, under medical follow-up or referred for AVR. It introduced new criteria using LVGLS and PALS for left-side damage and RVFWS for right-side damage to improve patient reclassification. Our staging system achieved a more balanced distribution of patients, especially in the earlier stages, with 34.4% and 21.3% classified in Stages 0 and 1, respectively, compared with the Généreux *et al*.^5^ and the Tastet *et al*.^10^ staging schemes. Each stage in our model was independently associated with both endpoints, even after adjusting for comorbidities and censoring for AVR. Patients in Stage 0 had the best outcomes, likely due to the absence of subclinical extravalvular damage. Outcomes worsened in Stage 1, where either LV or LA longitudinal deformation was impaired. Our findings underscore the prognostic value of LVGLS/PALS combination, with the worst prognosis observed in patients with impairments in both. The extent of damage to the right side of the heart was further classified according to RVFWS. Patients with preserved RVFWS had better outcomes than those with impaired RVFWS, including those with both LVGLS and PALS impairment. Unlike TAPSE and RV S′, which are affected by factors like angle dependency and load, RVFWS offers a more accurate assessment of myocardial function, better reflecting RV contraction physiology.^15^

### Clinical implications

Our staging system, incorporating LVGLS, PALS, and RVFWS, aids in risk stratification, potentially identifying moderate-to-severe AS patients for early intervention or closer monitoring. Emerging data suggest that some of these patients face a significant risk of mortality and myocardial damage.^25^ This proposed staging would potentially help them gain from interventions beyond the defined criteria suggested by guidelines.^17,18^

### Limitations

The study retrospectively analysed outpatients with significant AS referred to specialized tertiary university hospitals, potentially introducing selection bias. Although we excluded patients with a diagnosis of AS and confirmed cardiac amyloidosis, the retrospective design of our study did not permit a systematic evaluation for cardiac amyloidosis in the cohort of AS patients, as it was based solely on clinical and echocardiographic data.^26^ We measured LVGLS, PALS, and RVFWS with vendor-independent software, but results may not generalize to proprietary software packages. AF, which was prevalent in a substantial portion of the cohort, could have affected the accuracy of strain measurements. Additionally, 2D-STE parameters are influenced by both preload and afterload.^14,15^

## Conclusion

This multicentre study demonstrates that a novel staging classification that incorporates multi-chamber myocardial deformation assessed by 2D-STE can provide incremental prognostic value beyond previous staging systems used to assess the severity of AS. This novel staging model can help identify early cardiac dysfunction in patients with moderate and severe AS, which could help in early intervention or more stringent follow-up, especially in those at a higher risk.

## Supplementary Material

jeaf035_Supplementary_Data

## Data Availability

The data are available as appropriate request to the authors.

## References

[1] Osnabrugge RL , Mylotte D, Head SJ, Van Mieghem NM, Nkomo VT, LeReun CM et al Aortic stenosis in the elderly: disease prevalence and number of candidates for transcatheter aortic valve replacement: a meta-analysis and modeling study. J Am Coll Cardiol 2013;62:1002–12. 23727214 10.1016/j.jacc.2013.05.015

[2] Li SX , Patel NK, Flannery LD, Selberg A, Kandanelly RR, Morrison FJ et al Trends in utilization of aortic valve replacement for severe aortic stenosis. J Am Coll Cardiol 2022;79:864–77. 35241220 10.1016/j.jacc.2021.11.060

[3] Généreux P , Sharma RP, Cubeddu RJ, Aaron L, Abdelfattah OM, Koulogiannis KP et al The mortality burden of untreated aortic stenosis. J Am Coll Cardiol 2023;82:2101–9. 37877909 10.1016/j.jacc.2023.09.796

[4] Benfari G , Essayagh B, Michelena HI, Ye Z, Inojosa JM, Ribichini FL et al Severe aortic stenosis: secular trends of incidence and outcomes. Eur Heart J 2024;45:1877–86. 38190428 10.1093/eurheartj/ehad887

[5] Genereux P , Pibarot P, Redfors B, Mack MJ, Makkar RR, Jaber WA et al Staging classification of aortic stenosis based on the extent of cardiac damage. Eur Heart J 2017;38:3351–8. 29020232 10.1093/eurheartj/ehx381 PMC5837727

[6] Généreux P , Pibarot P, Redfors B, Bax JJ, Zhao Y, Makkar RR et al Evolution and prognostic impact of cardiac damage after aortic valve replacement. J Am Coll Cardiol 2022;80:783–800. 35595203 10.1016/j.jacc.2022.05.006

[7] Dahl JS , Magne J, Pellikka PA, Marwick DE, H T. Assessment of subclinical left ventricular dysfunction in aortic stenosis. JACC Cardiovasc Imaging 2019;12:163–71. 30621988 10.1016/j.jcmg.2018.08.040

[8] Medvedofsky D , Koifman E, Jarrett H, Miyoshi T, Rogers T, Ben-Dor I et al Association of right ventricular longitudinal strain with mortality in patients undergoing transcatheter aortic valve replacement. J Am Soc Echocardiogr 2020;33:452–60. 32033789 10.1016/j.echo.2019.11.014

[9] Springhetti P , Tomaselli M, Benfari G, Milazzo S, Ciceri L, Penso M et al Peak atrial longitudinal strain and risk stratification in moderate and severe aortic stenosis. Eur Heart J Cardiovasc Imaging 2024;25:947–57. 38319610 10.1093/ehjci/jeae040

[10] Tastet L , Tribouilloy C, Marechaux S, Vollema EM, Delgado V, Salaun E et al Staging cardiac damage in patients with asymptomatic aortic valve stenosis. J Am Coll Cardiol 2019;74:550–63. 31345430 10.1016/j.jacc.2019.04.065

[11] Lee CY , Nabeshima Y, Kitano T, Parasca CA, Calin A, Popescu BA et al Prognostic value of right ventricular free-wall longitudinal strain in aortic stenosis: a systematic review and meta-analysis. J Cardiol 2023;84:80–5. 38043709 10.1016/j.jjcc.2023.11.008

[12] Baumgartner HC , Hung JC-C, Bermejo J, Chambers JB, Edvardsen T, Goldstein S et al Recommendations on the echocardiographic assessment of aortic valve stenosis: a focused update from the European Association of Cardiovascular Imaging and the American Society of Echocardiography. Eur Heart J Cardiovasc Imaging 2017;18:254–75. 28363204 10.1093/ehjci/jew335

[13] Lang RM , Badano LP, Mor-Avi V, Afilalo J, Armstrong A, Ernande L et al Recommendations for cardiac chamber quantification by echocardiography in adults: an update from the American Society of Echocardiography and the European Association of Cardiovascular Imaging. J Am Soc Echocardiogr 2015;28:1–39.e14. 25559473 10.1016/j.echo.2014.10.003

[14] Voigt JU , Pedrizzetti G, Lysyansky P, Marwick TH, Houle H, Baumann R et al Definitions for a common standard for 2D speckle tracking echocardiography: consensus document of the EACVI/ASE/Industry Task Force to standardize deformation imaging. J Am Soc Echocardiogr 2015;28:183–93. 25623220 10.1016/j.echo.2014.11.003

[15] Badano LP , Kolias TJ, Muraru D, Abraham TP, Aurigemma G, Edvardsen T et al Standardization of left atrial, right ventricular, and right atrial deformation imaging using two-dimensional speckle tracking echocardiography: a consensus document of the EACVI/ASE/Industry Task Force to standardize deformation imaging. Eur Heart J Cardiovasc Imaging 2018;19:591–600. 29596561 10.1093/ehjci/jey042

[16] Dweck MR , Loganath K, Bing R, Treibel TA, McCann GP, Newby DE et al Multi-Modality imaging in aortic stenosis an EACVI clinical consensus document. Eur Heart J Cardiovasc Imaging 2023;24:1430–43. 37395329 10.1093/ehjci/jead153

[17] Otto CM , Nishimura RA, Bonow RO, Carabello BA, Erwin JP III, Gentile F et al 2020 ACC/AHA guideline for the management of patients with valvular heart disease: executive summary: a report of the American College of Cardiology/American Heart Association joint committee on clinical practice guidelines. Circulation 2021;143:e35–71. 33332149 10.1161/CIR.0000000000000932

[18] Vahanian A , Beyersdorf F, Praz F, Milojevic M, Baldus S, Bauersachs J et al 2021 ESC/EACTS guidelines for the management of valvular heart disease. Eur Heart J 2022;43:561–632. 34453165 10.1093/eurheartj/ehab395

[19] Stassen J , Pio SM, Ewe SH, Singh GK, Hirasawa K, Butcher SC et al Left ventricular global longitudinal strain in patients with moderate aortic stenosis. J Am Soc Echocardiogr 2022;35:791–800.e4. 35301093 10.1016/j.echo.2022.03.008

[20] Magne J , Cosyns B, Popescu BA, Carstensen HG, Dahl J, Desai MY et al Distribution and prognostic significance of left ventricular global longitudinal strain in asymptomatic significant aortic stenosis: an individual participant data meta-analysis. JACC Cardiovasc Imaging 2019;12:84–92. 30621997 10.1016/j.jcmg.2018.11.005

[21] Stens NA , van Iersel O, Rooijakkers MJP, van Wely MH, Nijveldt R, Bakker EA et al Prognostic value of preprocedural LV global longitudinal strain for post-TAVR-related morbidity and mortality: a meta-analysis. JACC Cardiovasc Imaging 2023;16:332–41. 36889849 10.1016/j.jcmg.2023.01.005

[22] Vollema EM , Amanullah MR, Prihadi EA, Ng ACT, van der Bijl P, Sin YK et al Incremental value of left ventricular global longitudinal strain in a newly proposed staging classification based on cardiac damage in patients with severe aortic stenosis. Eur Heart J Cardiovasc Imaging 2020;21:1248–58. 32851408 10.1093/ehjci/jeaa220

[23] Bohbot Y , Guignant P, Rusinaru D, Kubala M, Maréchaux S, Tribouilloy C. Impact of right ventricular systolic dysfunction on outcome in aortic stenosis. Circ Cardiovasc Imaging 2020;13:e009802. 31959010 10.1161/CIRCIMAGING.119.009802

[24] Ye Z , Yang LT, Medina-Inojosa JR, Scott CG, Padang R, Luis SA et al Multichamber strain characterization is a robust prognosticator for both bicuspid and tricuspid aortic stenosis. J Am Soc Echocardiogr 2022;35:956–65. 35613661 10.1016/j.echo.2022.05.010

[25] Stassen J , Ewe SH, Butcher SC, Amanullah MR, Hirasawa K, Singh GK et al Moderate aortic stenosis: importance of symptoms and left ventricular ejection fraction. Eur Heart J Cardiovasc Imaging 2022;23:790–9. 34864942 10.1093/ehjci/jeab242

[26] Abecasis J , Lopes P, Santos RR, Maltês S, Guerreiro S, Ferreira A et al Prevalence and significance of relative apical sparing in aortic stenosis: insights from an echo and cardiovascular magnetic resonance study of patients referred for surgical aortic valve replacement. Eur Heart J Cardiovasc Imaging 2023;24:1033–42. 36841934 10.1093/ehjci/jead032

