# Supplementary material for: A novel staging system of cardiac damage in aortic stenosis based on multi-chamber myocardial deformation
Source: Eur Heart J Cardiovasc Imaging. 2025 Jan 28;26(5):908–17. doi: 10.1093/ehjci/jeaf035 (PMC12042747; doi:10.1093/ehjci/jeaf035)
Supplement: jeaf035_Supplementary_Data [file jeaf035_supplementary_data.docx]

**SUPPLEMENTARY MATERIAL**

**Figure S1.** Patient selection flowchart of both the derivation cohort (Panel A) and the validation cohort (Panel B).

**

**

**Figure S2.** Pie charts showing the distribution of the patients included in the derivation cohort according to the staging systems proposed by Généreux et al.[5] (Panel A) and Tastet et al.[10] (Panel B).

**
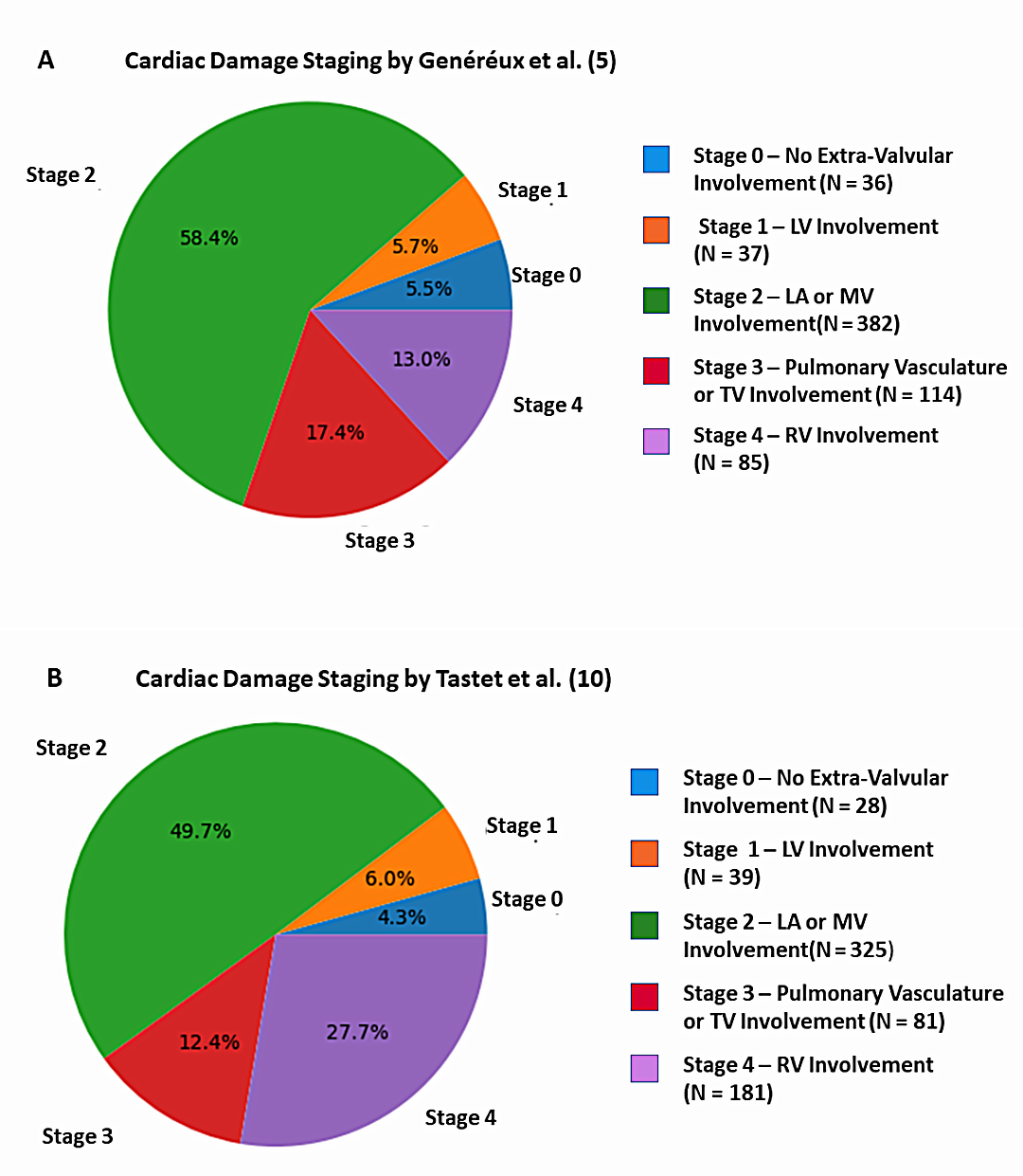
**

**Figure S3.** Kaplan-Meier analyses for all-cause mortality (Panel A) and all-cause mortality plus hospitalization for heart failure (Panel B) based on the Généreux et al.[5] staging system. Similar analyses, censored at the time of aortic valve replacement, are shown for all-cause mortality (Panel C) and all-cause mortality plus hospitalization for heart failure (Panel D). *Abbreviations: as in Central Illustration*

**
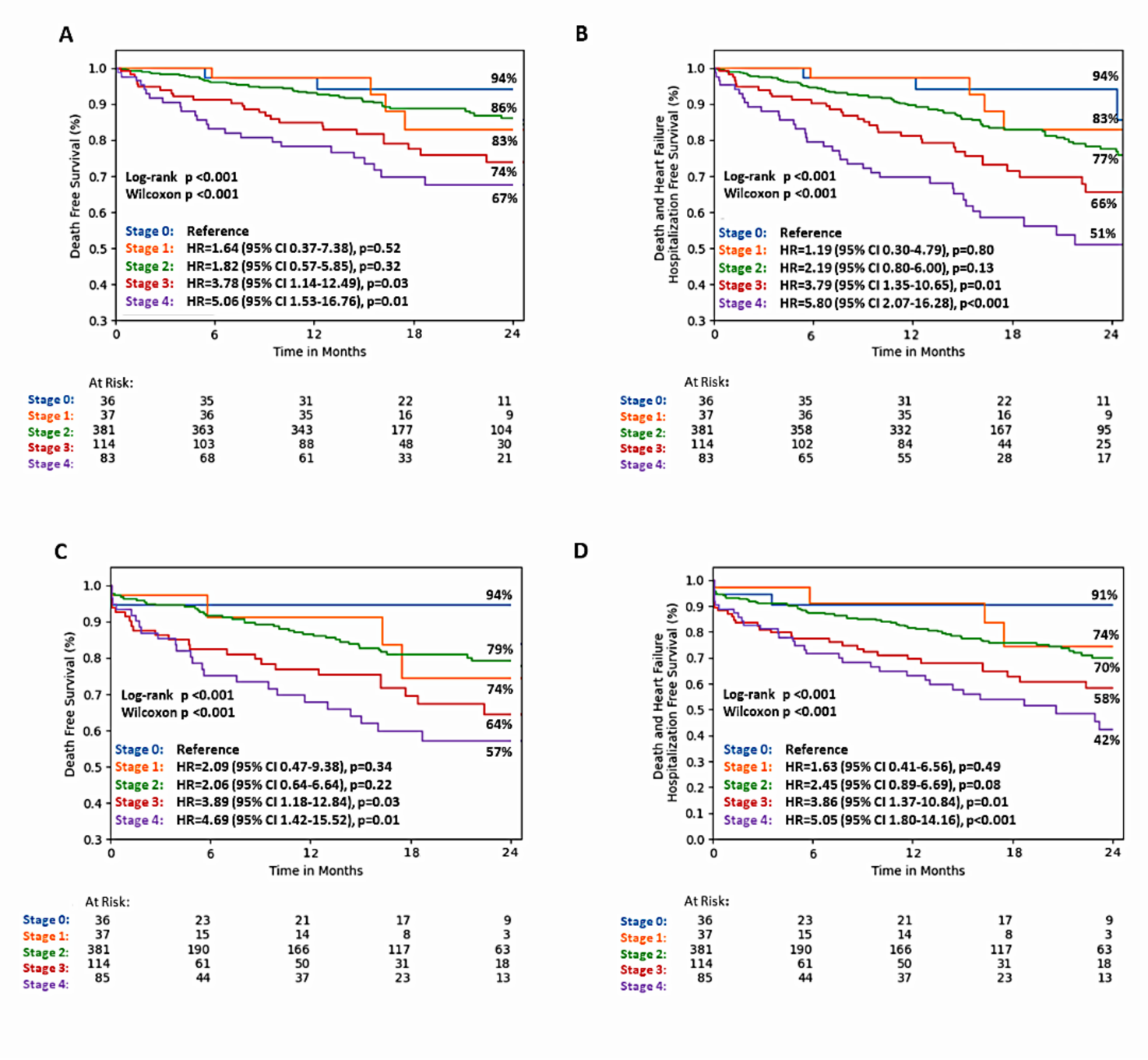
**

**Figure S4.** Kaplan-Meier analyses for all-cause mortality (Panel A) and all-cause mortality plus hospitalization for heart failure (Panel B) based on the Tastet et al.[10] staging system. Similar analyses, censored at the time of aortic valve replacement, are shown for all-cause mortality (Panel C) and all-cause mortality plus hospitalization for heart failure (Panel D). *Abbreviations as in Central Illustration.*

**
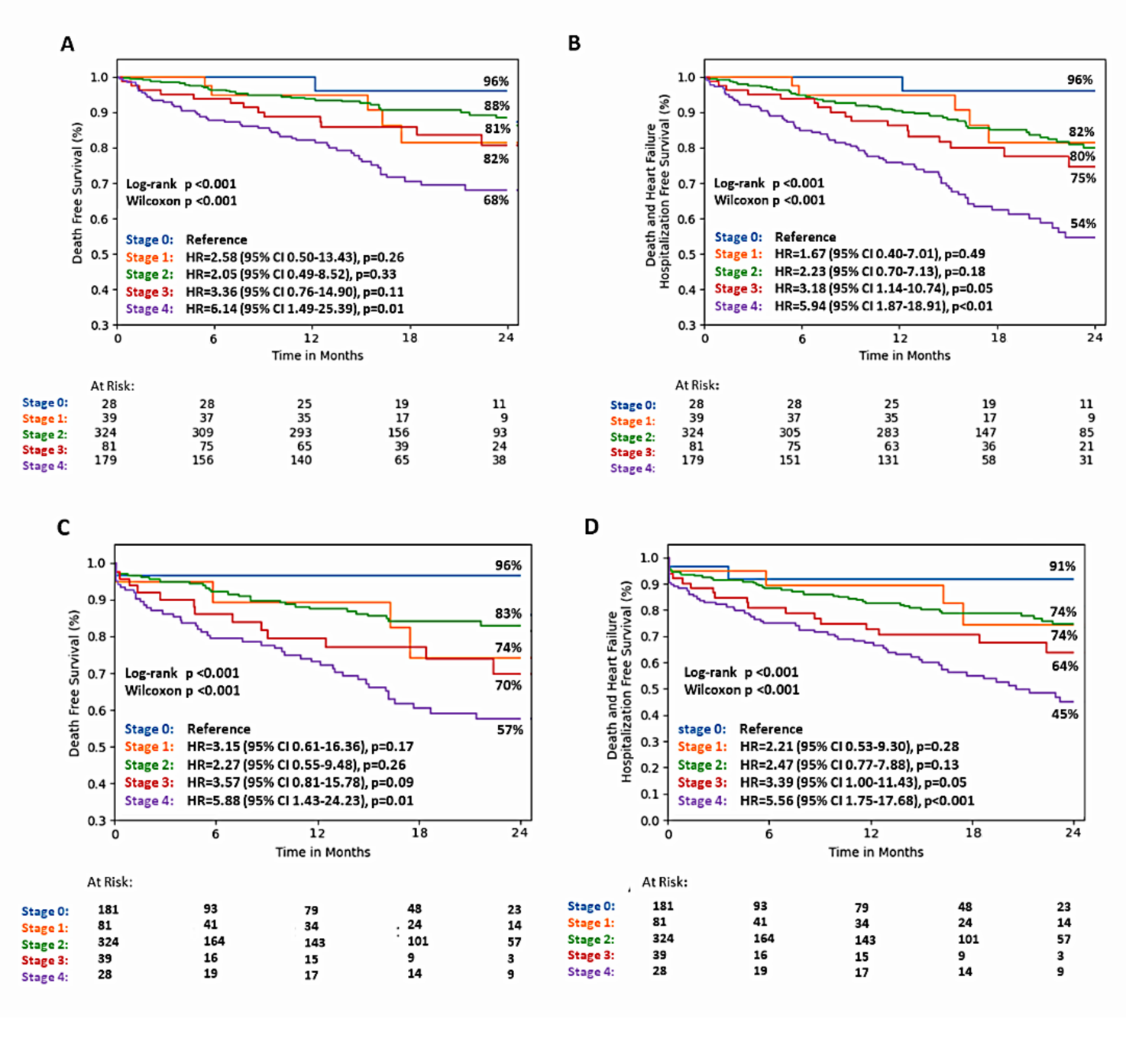
**

**Figure S5.** Marginal effects analysis showing the predicted probabilities (and their 9%% confidence interval) of 2-year mortality (Panel A) and composite endpoint (Panel B) using the proposed staging model.

**

**

**Figure S6.** Hierarchical-χ² analysis for primary (Panel A) and secondary (Panel B) endpoints. Model 0 included aortic valve area, left ventricular mass index, and left ventricular ejection fraction. Model 1 expanded on Model 0 by adding left atrial volume index and ≥ moderate mitral regurgitation. Model 2 further incorporated TAPSE, pulmonary artery systolic pressure, and ≥ moderate tricuspid regurgitation. Finally, Model 3 included the proposed staging system as an additional variable.


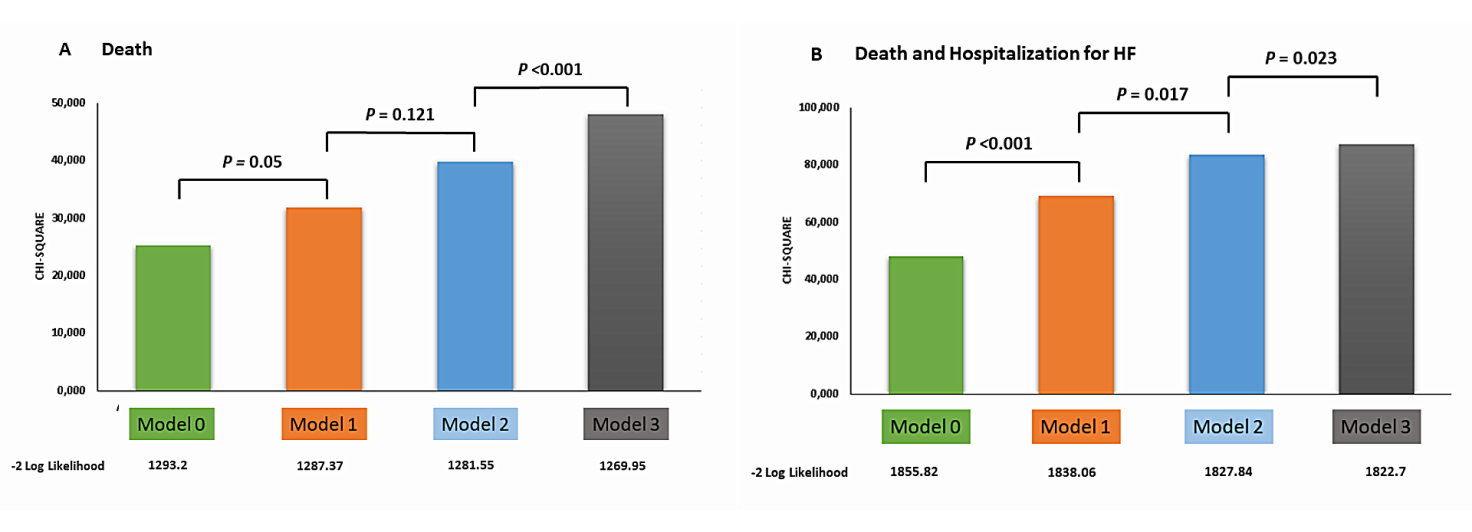


**Figure S7**. Kaplan–Meier analyses for all-cause mortality (Panel A) and all-cause mortality plus hospitalization for heart failure (Panel B) according to the proposed staging scheme in the validation cohort. The time-dependent ROC curves were utilized to compare the proposed and the previously reported staging models for the primary (Panel C) and secondary (Panel D) endpoints at 3 years in the validation cohort.

**
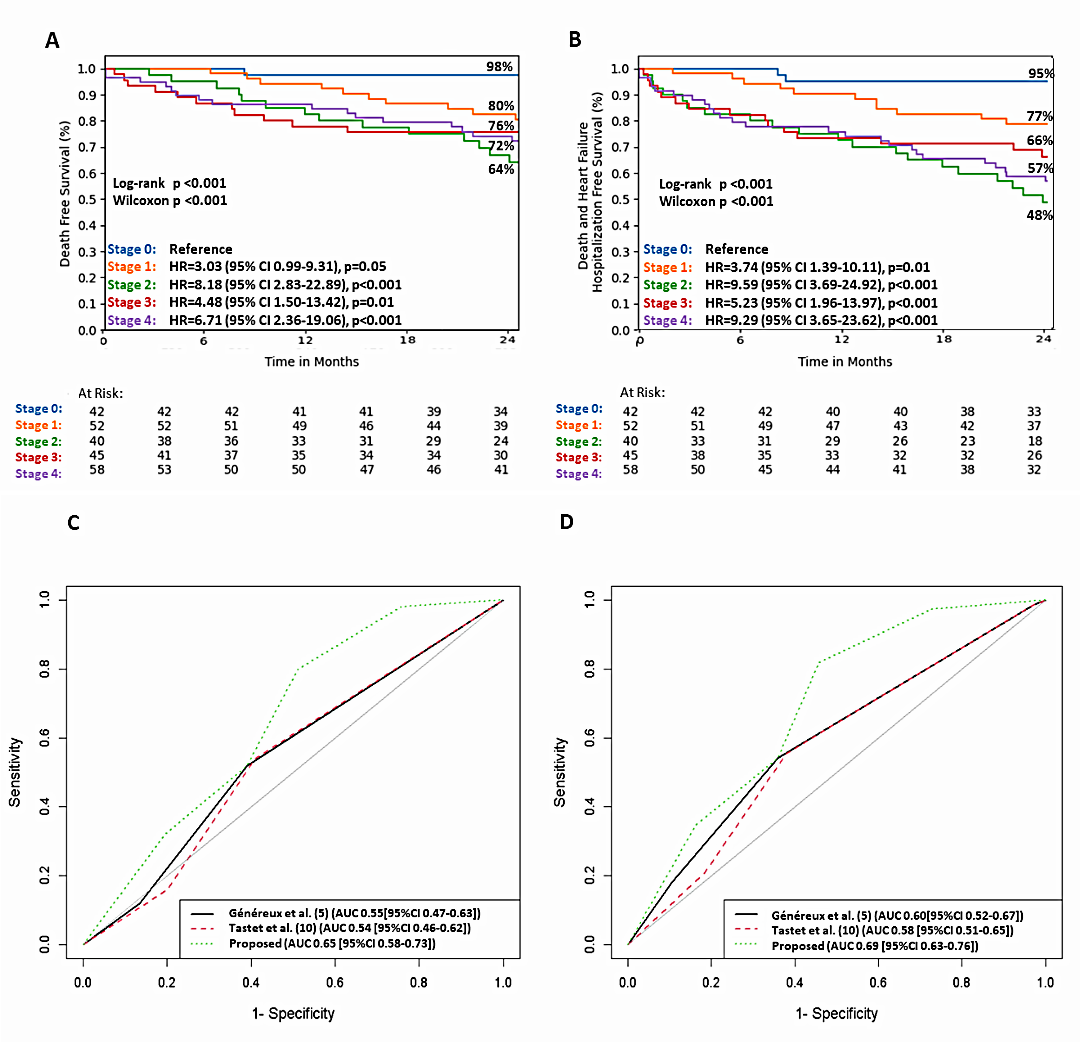
**

**Table S1.** Cardiac stratification of AS based on the extent of cardiac damage according to Généreux et al.[5] and Tastet et al.[10]

|  | **Stage 0**  **No Cardiac Damage** | **Stage 1**  **Left Ventricular Damage** | **Stage 2**  **Left Atrial or Mitral Valve Damage** | **Stage 3**  **Pulmonary Vasculature or Tricuspid Valve Damage** | **Stage 4**  **Right Ventricular Damage** |
| --- | --- | --- | --- | --- | --- |
| Généreux *et al.[1]* | No cardiac damage | LV hypertrophy (>95 g/m^2^ women; >115 g/m^2^ men)  E/e’ ratio > 14  LV ejection fraction < 50% | Left atrial volume >34 ml/m^2^  MR ≥ moderate  Atrial fibrillation | Pulmonary hypertension (PASP ≥60 mm Hg)  TR≥ moderate | RV systolic dysfunction ≥ moderate |
| *Tastet et al.[2]* | No cardiac damage | LV hypertrophy (>95 g/m^2^ women; >115 g/m^2^ men)  LV diastolic dysfunction > grade II  LV GLS ≤15%  LV ejection fraction <60% | Left atrial volume >34 ml/m^2^  MR ≥ moderate  Atrial fibrillation | Pulmonary hypertension (PASP ≥60 mm Hg)  TR≥ moderate | RV systolic dysfunction ≥ moderate (TAPSE < 17 mm, S’ TDI < 9.5 cm/s)  Moderate to severe low-flow (LV SVi <30 ml/m^2^) |

*Abbreviations: GLS, global longitudinal strain; LV, left ventricular; MR, mitral regurgitation; PASP, pulmonary artery systolic pressure; RV, right ventricular; SVi, stroke volume indexed; TR, tricuspid regurgitation.*

**Table S2.** Baseline Characteristics of the patients included in the derivation and validation cohorts

|  | **Derivation**  **(n = 654)** | **Validation**  **(n = 237)** | **p** |
| --- | --- | --- | --- |
| Demographics |  |  |  |
| Age, years | 81 (81-82) | 77 (71-81) | **<0.001** |
| Male | 336(51) | 130(55) | 0.100 |
| Body surface area (m^2^) | 1.78 (1.76-1.79) | 1.82 (1.67-1.95) | **0.017** |
| Body mass index (kg/m^2^) | 26.1 (25.8-26.5) | 27.4 (24.3-30.8) | **<0.001** |
| Cardiovascular risk factors |  |  |  |
| Smoking, n(%) | 155(24) | 30(13) | **<0.001** |
| Hypertension, n(%) | 395(60) | 201(85) | **<0.001** |
| Diabetes mellitus, n(%) | 176(27) | 60(25) | 0.600 |
| Dyslipidemia, n(%) | 370(57) | 209(88) | **<0.001** |
| NYHA functional class ≥III, n(%) | 219(33) | 149(63) | **<0.001** |
| Angina, n(%) | 115(18) | 78(33) | **<0.001** |
| Syncope, n(%) | 59(9) | 16(7) | 0.268 |
| Coronary artery disease, n(%) | 217(33) | 107(45) | **0.001** |
| Estimated glomerular filtration rate (mL/min/1.73 m^2^) | 55 (53-57) | 61 (46-78) | **<0.001** |
| Systolic blood pressure (mmHg) | 137 (135-138) | 120 (106-138) | **<0.001** |
| Diastolic blood pressure (mmHg) | 76 (75-77) | 70 (60-80) | **<0.001** |
| Heart rhythm at the time of TTE |  |  | 0.477 |
| Sinus rhythm | 511(78) | 180 (76) |  |
| Atrial fibrillation | 143(22) | 57 (24) |  |
| Aortic stenosis |  |  | **<0.001** |
| Moderate | 258(39) | 15(6) |  |
| Severe | 396(61) | 222(94) |  |
| TTE characteristics |  |  |  |
| Relative wall thickness | 0.53 (0.50-0.55) | 0.61 (0.49-0.72) | **<0.001** |
| Septal wall thickness (cm) | 1.3 (1.2-1.3) | 1.5 (1.3-1.7) | **<0.001** |
| Posterior wall thickness (cm) | 1.1 (1.1-1.2) | 1.4 (1.2-1.6) | **<0.001** |
| LVED diameter (cm) | 4.6 (4.5-4.7) | 4.7 (4.1-5.2) | 0.118 |
| LV mass index (g/m^2^) | 114 (112-117) | 155 (124-194) | **<0.001** |
| LVEDV (mL/m^2^) | 63 (61-64) | 43 (33-57) | **<0.001** |
| LVESV (mL/m^2^) | 28 (17-30) | 27 (19-35) | 0.006 |
| LVSV (mL/m^2^) | 37 (36-38) | 44 (36-54) | **<0.001** |
| LVEF (%) | 57.0 (56.0-57.9) | 50.5 (41.9-56.6) | **<0.001** |
| LAV (mL/m^2^) | 46 (38-58) | 36 (21-53) | **<0.001** |
| Average E/e’ ratio | 14.1 (13.8-14.5) | 9.7 (0.3-17.4) | **<0.001** |
| MR≥ moderate | 188(29) | 59(25) | 0.256 |
| Systolic pulmonary arterial pressure (mmHg) | 39 (38-40) | 37 (29-51) | 0.588 |
| TR ≥ moderate | 140(21) | 79(33) | **<0.001** |
| Mean aortic valve gradient (mmHg) | 31.6 (30.3-32.9) | 52.2 (42.8-65.2) | **<0.001** |
| Aortic valve area (cm^2^) | 0.86 (0.84-0.88) | 0.74 (0.57-0.90) | **<0.001** |
| LV global LS, % | 14.0 (13.7-14.4) | 13.0 (9.4-16.3) | **0.001** |
| RV free-wall LS, % | 20.9 (20.4-21.4) | 21.7 (15.8-25.8) | 0.441 |
| Peak atrial LS, % | 17.7 (16.9-18.4) | 11.2 (7.3-17.6) | **<0.001** |

Values are mean (95% CI) or n(%).

Abbreviations: CKD, chronic kidney disease; EDV, end-diastolic volume; ESV, end-systolic volume; LAV, left atrial volume; LV, left ventricle/ventricular, LVEF, left ventricular ejection fraction; LS, longitudinal strain; MR, mitral regurgitation; RV right ventricle/ventricular; SV, stroke volume; TR, tricuspid regurgitation.

**Table S3.** Staging of the Derivation Cohort According to the Généreux et al.[5] Classification.

|  | **Stage 0**  **(n = 36)** | **Stage 1**  **(n = 37)** | **Stage 2**  **(n = 382)** | **Stage 3**  **(n = 114)** | **Stage 4**  **(n = 85)** | ***p* value** |
| --- | --- | --- | --- | --- | --- | --- |
| Demographics |  |  |  |  |  |  |
| Age, years | 74.8 (70.7-78.9) | 77.2 (74.2-80.3) | 81.4 (80.7-82.2)†‡ | 83.9 (82.7-85.0)†‡§ | 83.5 (82.0-84.9)†‡§ | **<0.001** |
| Male, n(%) | 22(61) | 19(51) | 191(50) | 56(49) | 48(56) | 0.597 |
| Body surface area (m^2^) | 1.80 (1.73-1.88) | 1.80 (1.74-1.86) | 1.78 (1.76-1.80) | 1.76 (1.72-1.80) | 1.76 (1.71-1.80) | 0.674 |
| Body mass index (kg/m^2^) | 26.5 (24.6-28.3) | 27.1 (25.5-28.7) | 26.2 (25.7-26.7) | 25.6 (24.8-26.5) | 25.8 (24.7-26.8) | 0.611 |
| Cardiovascular risk factors |  |  |  |  |  |  |
| Smoking, n(%) | 6(17) | 15(40) | 98(26) | 20(17) | 16(19) | **0.025** |
| Hypertension, n(%) | 23(64) | 25(68) | 230(61) | 74(65) | 43(51) | 0.230 |
| Diabetes mellitus, n(%) | 9(26) | 14(38) | 109(29) | 23(20) | 21(25) | 0.237 |
| Dyslipidemia, n(%) | 25(69) | 25(68) | 212(56) | 63(56) | 45(53) | 0.316 |
| NYHA functional class ≥III, n(%) | 4(11) | 8(22) | 114(30) | 49(44) | 44(52) | **<0.001** |
| Angina, n(%) | 6(17) | 8(22) | 72(19) | 22(20) | 7(8) | 0.181 |
| Syncope, n(%) | 2(6) | 4(11) | 35(9) | 10(9) | 8(9) | 0.951 |
| COPD, n(%) | 2(6) | 4(11) | 29(8) | 13(11) | 9(11) | 0.615 |
| Coronary artery disease, n(%) | 10(28) | 16(43) | 115(30) | 38(34) | 38(45) | 0.069 |
| Previous myocardial infarction, n(%) | 6(17) | 8(22) | 54(14) | 24(21) | 30(35) | **<0.001** |
| Previous acute heart failure, n(%) | 0(0) | 2(5) | 60(16) | 37(33) | 30(36) | **<0.001** |
| eGFR (mL/min/m^2^) | 70 (58-81) | 66 (53-78) | 56 (54-59)† | 48 (44-53)†‡§ | 49 (44-54)†‡§ | **<0.001** |
| Systolic blood pressure (mm Hg) | 137 (129-144) | 142 (135-148) | 139 (137-141) | 134 (130-138)§ | 125 (120-130)†‡§\|\| | **<0.001** |
| Diastolic blood pressure (mm Hg) | 77 (73-82) | 77 (73-81) | 76 (75-77) | 75 (73-77) | 73 (71-75) | 0.109 |
| Heart rhythm at the time of TTE |  |  |  |  |  | **<0.001** |
| Sinus rhythm, n(%) | 36(100) | 37(100) | 325(85) | 73(64) | 40(47) |  |
| Atrial fibrillation, n(%) | 0(0) | 0(0) | 57(15) | 41(36) | 45(53) |  |
| Aortic stenosis |  |  |  |  |  | 0.328 |
| Moderate, n(%) | 20(56) | 15(40) | 143(37) | 46(40) | 34(40) |  |
| Severe, n(%) | 16(44) | 22(60) | 239(63) | 68(60) | 51(60) |  |
| TTE characteristics |  |  |  |  |  |  |
| Relative wall thickness | 0.50 (0.46-0.54) | 0.66 (0.38-0.94) | 0.54 (0.50-0.58) | 0.49 (0.46-0.52) | 0.49 (0.46-0.52) | 0.245 |
| Septal wall thickness, cm | 1.2 (1.1-1.2) | 1.3 (1.2-1.5)† | 1.3 (1.2-1.3)† | 1.2 (1.2-1.3) | 1.2 (1.2-1.3) | **0.036** |
| Posterior wall thickness, cm | 1.0 (1.0-1.0) | 1.1 (1.0-1.2)† | 1.1 (1.1-1.2)† | 1.1 (1.0-1.2)† | 1.2 (1.0-1.3)† | **0.005** |
| LV end-diastolic diameter, cm | 4.2 (4.0-4.4) | 4.4 (4.1-4.7) | 4.6 (4.5-4.6)† | 4.8 (4.4-5.2)† | 4.9 (4.7-5.0)†‡§ | **<0.001** |
| LV mass, g/m^2^ | 84 (78-91) | 108 (100-116)† | 114 (111-118)† | 116 (109-122)† | 126 (117-136)†‡§ | **<0.001** |
| LV end-diastolic volume, mL/m^2^ | 57 (54-61) | 54 (50-59) | 63 (61-65)†‡ | 61 (57-65)§ | 69 (64-75)†‡\|\| | **0.002** |
| LV end-systolic volume, mL/m^2^ | 22 (20-23) | 22 (19-25) | 27 (25-28)‡ | 29 (25-32) | 41 (35-46)†‡§\|\| | **<0.001** |
| LV stroke volume, mL/m^2^ | 40 (37-43) | 38 (36-40) | 39 (38-40) | 35 (33-37)†‡§ | 29 (27-30)†‡§\|\| | **<0.001** |
| LV ejection fraction, % | 62.2 (60.8-63.6) | 62.1 (59.1-65.0) | 58.9 (57.9-60.0) | 55.9 (53.6-58.1)†‡§ | 45.2 (41.8-48.5)†‡§\|\| | **<0.001** |
| LV ejection fraction<50%, n(%) | 0(0) | 4(11) | 64(17) | 31(27) | 47(55) | **<0.001** |
| Left atrial volume, mL/m^2^ | 29 (28-31) | 29 (28-30) | 49 (47-50)†‡ | 59 (56-63)†‡§ | 59 (55-63)†‡§ | **<0.001** |
| E/e’ ratio | 9.4 (8.7-10.1) | 12.9 (11.7-14.1)† | 13.8 (13.4-14.3)† | 15.9 (14.9-16.8)†‡§ | 15.7 (14.6-16.8)†‡§ | **<0.001** |
| MR≥ moderate, n(%) | 0(0) | 0(0) | 73(19) | 69(60) | 46(54) | **<0.001** |
| PASP, mm Hg | 28.4 (27.3-29.6) | 31.3 (29.4-33.1)† | 35.4 (34.5-36.2)†‡ | 51.7 (49.3-54.2)†‡§ | 46.4 (43.2-49.7)†‡§\|\| | **<0.001** |
| TR ≥ moderate, n(%) | 0(0) | 0(0) | 0(0) | 95(83) | 45(53) | **<0.001** |
| Mean aortic valve gradient, mm Hg | 29.7 (24.8-34.7) | 33.7 (29.0-38.5) | 34.5 (32.8-36.3) | 28.5 (25.6-31.3)‡§ | 22.7 (19.9-25.5)†‡§\|\| | **<0.001** |
| Aortic valve area, cm^2^ | 0.97 (0.87-1.06) | 0.85 (0.77-0.94) | 0.89 (0.86-0.92) | 0.88 (0.83-0.93) | 0.90 (0.82-0.98) | 0.453 |

Values are mean (95% CI) or n(%). *The p values depict differences between stages of extra-aortic valvular cardiac abnormalities: †p < 0.05 vs. Group 0 with Bonferroni’s post hoc analysis. ‡p < 0.05 vs. Group 1 with Bonferroni’s post hoc analysis. §p < 0.05 vs. Group 2 with Bonferroni’s post hoc analysis. ||p < 0.05 vs. Group 3 with Bonferroni’s post hoc analysis.

*Abbreviations*: COPD, chronic obstructive pulmonary disease; GFR, glomerular filtration rate; LV, left ventricle/ventricular; MR, mitral regurgitation; PASP, pulmonary artery systolic pressure; RV right ventricle/ventricular; TR, tricuspid regurgitation.

**Table S4.** Staging of the Derivation Cohort According to the Tastet et al.[10] Classification.

|  | **Stage 0**  **(n = 28)** | **Stage 1**  **(n = 39)** | **Stage 2**  **(n = 325)** | **Stage 3**  **(n = 81)** | **Stage 4**  **(n = 181)** | ***p* value** |
| --- | --- | --- | --- | --- | --- | --- |
| Demographics |  |  |  |  |  |  |
| Age, years | 73.6 (68.9-78.4) | 77.3 (74.3-80.3) | 81.4 (80.6-82.2)†‡ | 83.4 (82.0-84.8)†‡ | 83.0 (82.1-84.0)†‡ | **<0.001** |
| Male, n(%) | 19(68) | 21(54) | 161(50) | 38(47) | 97(54) | 0.333 |
| Body surface area (m^2^) | 1.82 (1.73-1.90) | 1.79 (1.72-1.85) | 1.78 (1.75-1.80) | 1.76 (1.71-1.80) | 1.78 (1.75-1.81) | 0.847 |
| Body mass index (kg/m^2^) | 26.8 (24.5-29.0) | 26.7 (25.2-28.2) | 26.3 (25.7-26.8) | 26.0 (25.0-27.0) | 25.7 (25.0-26.4) | 0.622 |
| Cardiovascular risk factors |  |  |  |  |  |  |
| Smoking, n(%) | 6(21) | 15(39) | 82(25) | 16(20) | 36(20) | 0.118 |
| Hypertension, n(%) | 18(64) | 26(67) | 196(60) | 53(65) | 105(58) | 0.725 |
| Diabetes mellitus, n(%) | 7(26) | 15(39) | 91(28) | 13(16) | 50(28) | 0.109 |
| Dyslipidemia, n(%) | 21(75) | 27(69) | 185(58) | 46(58) | 91(50) | 0.050 |
| NYHA functional class ≥III, n(%) | 3(11) | 9(24) | 89(28) | 29(38) | 89(49) | **<0.001** |
| Angina, n(%) | 5(18) | 8(21) | 63(20) | 17(22) | 22(12) | 0.231 |
| Syncope, n(%) | 1(4) | 4(10) | 31(10) | 6(8) | 17(9) | 0.838 |
| COPD, n(%) | 1(4) | 4(10) | 26(8) | 11(14) | 15(8) | 0.448 |
| Coronary artery disease, n(%) | 9(32) | 16(41) | 104(32) | 24(30) | 64(35) | 0.740 |
| Previous myocardial infarction, n(%) | 5(18) | 8(21) | 47(15) | 17(21) | 45(25) | 0.072 |
| Previous acute heart failure, n(%) | 0(0) | 2(5) | 51(16) | 23(30) | 53(29) | **<0.001** |
| eGFR (mL/min/m^2^) | 69 (59-78) | 65 (55-74) | 57 (54-59)† | 49 (43-54)†‡§ | 50 (48-53)†‡§ | **<0.001** |
| Systolic blood pressure (mm Hg) | 137 (129-145) | 141 (136-147) | 104 (138-142) | 137 (133-141) | 130 (127-133)‡§\|\| | **<0.001** |
| Diastolic blood pressure (mm Hg) | 78 (73-83) | 77 (73-81) | 76 (74-77) | 75 (73-78) | 75 (73-77) | 0.701 |
| Heart rhythm at the time of TTE |  |  |  |  |  | **<0.001** |
| Sinus rhythm, n(%) | 28(100) | 39(100) | 289(89) | 56(69) | 99(55) |  |
| Atrial fibrillation, n(%) | 0(0) | 0(0) | 36(11) | 25(31) | 82(45) |  |
| Aortic stenosis |  |  |  |  |  | 0.236 |
| Moderate, n(%) | 17(61) | 15(39) | 125(39) | 31(38) | 70(39) |  |
| Severe, n(%) | 11(39) | 24(62) | 200(62) | 50(62) | 111(61) |  |
| TTE characteristics |  |  |  |  |  |  |
| Relative wall thickness | 0.49 (0.45-0.53) | 0.53 (0.49-0.58) | 0.54 (0.49-0.58) | 0.48 (0.45-0.52) | 0.53 (0.48-0.58) | 0.358 |
| Septal wall thickness, cm | 1.2 (1.1-1.2) | 1.3 (1.2-1.3) | 1.3 (1.2-1.3) | 1.3 (1.2-1.3) | 1.3 (1.2-1.3) | 0.118 |
| Posterior wall thickness, cm | 1.0 (1.0-1.1) | 1.1 (1.0-1.1) | 1.1 (1.1-1.2) | 1.2 (1.0-1.3) | 1.1 (1.1-1.2) | 0.087 |
| LV end-diastolic diameter, cm | 4.3 (4.1-4.5) | 4.4 (4.1-4.6) | 4.6 (4.5-4.6)† | 5.0 (4.4-5.5)†‡ | 4.6 (4.5-4.8) | **0.031** |
| LV mass, g/m^2^ | 87 (79-95) | 106 (97-114)† | 115 (111-118)† | 120 (112-129)†‡ | 117 (111-123)† | **<0.001** |
| LV end-diastolic volume, mL/m^2^ | 59 (55-63) | 55 (51-59) | 64 (62-66)‡ | 63 (59-68) | 63 (59-66)§ | **0.002** |
| LV end-systolic volume, mL/m^2^ | 22 (21-24) | 22 (19-25) | 26 (25-28)‡ | 29 (25-32) | 35 (31-38)‡§ | **0.025** |
| LV stroke volume, mL/m^2^ | 42 (39-45) | 38 (36-40)† | 41 (40-42)‡ | 39 (37-41)†§ | 27 (27-28)†‡§\|\| | **<0.001** |
| LV ejection fraction, % | 61.9 (60.2-63.5) | 62.6 (60.0-65.2) | 60.3 (59.2-61.3) | 57.3 (54.8-59.8)‡§ | 49.0 (47.0-51.1)†‡§\|\| | **<0.001** |
| LV ejection fraction<50%, n(%) | 0(0) | 3(8) | 42(13) | 18(22) | 83(46) | **<0.001** |
| Left atrial volume, mL/m^2^ | 29 (28-31) | 29 (28-30) | 49 (47-50)†‡ | 60 (55-64)†‡§ | 55 (53-58)†‡§ | **<0.001** |
| E/e’ ratio | 9.7 (8.8-10.5) | 12.6 (11.4-13.8)† | 13.9 (13.4-14.4)† | 16.6 (15.5-17.7)†‡§ | 14.5 (13.8-15.3)†‡\|\| | **<0.001** |
| MR≥ moderate, n(%) | 0(0) | 0(0) | 63(20) | 47(58) | 78(43) | **<0.001** |
| PASP, mm Hg | 28.6 (27.3-29.9) | 30.8 (29.1-32.6)† | 35.2 (34.3-36.1)†‡ | 52.2 (49.6-54.8)†‡§ | 43.4 (41.3-45.6)†‡§\|\| | **<0.001** |
| TR ≥ moderate, n(%) | 0(0) | 0(0) | 0(0) | 65(80) | 75(41) | **<0.001** |
| Mean aortic valve gradient, mmHg | 29.2 (23.7-34.6) | 34.7 (29.9-39.5) | 35.4 (33.5-37.4) | 30.8 (27.4-34.2) | 24.9 (22.9-26.9)‡§\|\| | **<0.001** |
| Aortic valve area, cm^2^ | 1.02 (0.92-1.13) | 0.82 (0.75-0.89)† | 0.87 (0.84-0.90)† | 0.85 (0.80-0.90)† | 0.84 (0.79-0.88)† | **0.004** |
| LV global LS, % | 19.1 (18.4-19.8) | 15.3 (14.2-16.4)† | 15.4 (15.0-15.8)† | 13.9 (13.0-14.8)†§ | 10.6 (9.99-11.2)†‡§\|\| | **<0.001** |

Values are mean (95% CI) or n(%). *The p values depict differences between stages of extra-aortic valvular cardiac abnormalities: †p < 0.05 vs. Group 0 with Bonferroni’s post hoc analysis. ‡p < 0.05 vs. Group 1 with Bonferroni’s post hoc analysis. §p < 0.05 vs. Group 2 with Bonferroni’s post hoc analysis. ||p < 0.05 vs. Group 3 with Bonferroni’s post hoc analysis.

*Abbreviations*: COPD, chronic obstructive pulmonary disease; GFR, glomerular filtration rate; LV, left ventricle/ventricular; LS, longitudinal strain; MR, mitral regurgitation; PASP, pulmonary artery systolic pressure; RV right ventricle/ventricular; TR, tricuspid regurgitation.

**Table S5.** Clinical outcomes at various stages of cardiac damage.

|  | **Stage 0**  **(n = 225)** | **Stage 1**  **(n = 139)** | **Stage 2**  **(n = 91)** | **Stage 3**  **(n = 75)** | **Stage 4**  **(n = 124)** | ***p* value** |
| --- | --- | --- | --- | --- | --- | --- |
| Surgical or transcatheter AVR, n(%) | 110(49) | 79(57) | 51(56) | 24(32) | 61(49) | **0.008** |
| Time to surgical or transcatheter AVR, (days) | 375 (2-1842) | 306 (2-1726) | 238 (3-989) | 412 (2-1008) | 289 (3-1793) |  |
| All-cause death, n(%) | 16(7) | 17(12) | 23(25) | 16(21) | 39(32) | **<0.001** |
| Time to death, (days) | 631 (24-2969) | 580 (61-1805) | 492 (7-1157) | 549 (26-1236) | 527 (5-1793) |  |
| Hospitalization for HF, n(%) | 10(4) | 14(10) | 9(10) | 4(5) | 25(20) | **<0.001** |
| Time to hospitalization for HF, (days) | 621 (6-2969) | 540 (17-1258) | 470 (7-1094) | 538 (26-1236) | 476 (2-1793) |  |
| Combined endpoint (death and hospitalization for HF) , n(%)† | 26(12) | 30(22) | 30(33) | 19(25) | 57(46) | **<0.001** |
| Time to the combined endpoint, (days) | 621 (6-2969) | 553 (60-1805) | 473 (7-1094) | 538 (26-1236) | 482 (2-1793) |  |

Values are n(%) or mean (range). †For the secondary outcome, patients were censored at the occurrence of the first event. Time is expressed in days. Abbreviations: AVR, aortic valve replacement; HF, heart failure.

**Table S6.** Net reclassification improvement (NRI) and integrated discrimination improvement(IDI) analysis compared to previous classifications of extra-valvular cardiac damage at the 2-year follow-up in the derivation cohort.

| **Primary endpoint (all-cause death)** | | | | |
| --- | --- | --- | --- | --- |
|  | **Index** | **Estimate** | **95% CI** | ***p* value** |
| Proposed staging vs. Généreux et al.[5] | NRI | 0.281 | 0.033 – 0.378 | 0.045 |
| Proposed staging vs. Généreux et al.[5] | IDI | 0.027 | 0.001 – 0.051 | 0.02 |
| Proposed staging vs. Tastet et al.[10] | NRI | 0.145 | 0.017 – 0.287 | 0.045 |
| Proposed staging vs. Tastet et al.[10] | IDI | 0.010 | -0.021 – 0.041 | 0.437 |
| **Secondary endpoint (all-cause death and hospitalization for HF)** | | | | |
|  | **Index** | **Estimate** | **95% CI** | ***p* value** |
| Proposed staging vs. Généreux et al.[5] | NRI | 0.1516 | 0.0155 – 0.3186 | 0.075 |
| Proposed staging vs. Généreux et al.[5] | IDI | 0.0302 | 0.0137 – 0.0467 | <0.001 |
| Proposed staging vs. Tastet et al.[10] | NRI | 0.3251 | 0.1499 – 0.5002 | <0.001 |
| Proposed staging vs. Tastet et al.[10] | IDI | 0.0277 | 0.0102 – 0.0452 | 0.002 |

**Table S7.** Association of the proposed staging scheme with all-cause mortality and the combined endpoint (mortality and hospitalization for heart failure) in the derivation cohort patients with severe AS.

|  | **Univariable** | | **Multivariable** | |
| --- | --- | --- | --- | --- |
|  | **HR (95% CI)** | ***p* value** | **HR (95% CI)** | ***p* value** |
| All-cause mortality |  |  |  |  |
| Age, years | 1.10 (1.05-1.16) | <0.001 | 1.04 (0.98-1.09) | 0.151 |
| Male | 0.85 (0.52-1.41) | 0.549 | 1.05 (0.60-1.81) | 0.857 |
| Previous myocardial infarction | 1.58 (0.89-2.79) | 0.111 | 1.15 (0.45-2.89) | 0.761 |
| Atrial fibrillation | 1.44 (0.84-2.48) | 0.181 | 0.64 (0.35-1.19) | 0.167 |
| NYHA functional class ≥III | 1.70 (1.03-2.82) | 0.037 | 1.40 (0.80-2.43) | 0.229 |
| eGFR (per 1 mL/min/1.73 m^2^ increase) | 0.97 (0.95-0.98) | <0.001 | 0.99 (0.97-1.01) | 0.586 |
| Hypertension | 3.08 (1.77-5.34) | <0.001 | 2.03 (1.033-3.99) | **0.040** |
| Diabetes mellitus | 1.18 (0.69-2.02) | 0.540 | 1.16 (0.63-2.14) | 0.618 |
| Aortic valve area (per 1 cm^2^ increase) | 0.52 (0.13-2.08) | 0.363 | 0.40 (0.09-1.76) | 0.229 |
| Surgical or transcatheter AVR | 0.16 (0.09-0.28) | <0.001 | 0.26 (0.13-0.51) | **<0.001** |
| Stage of cardiac damage (by each stage increase) | 1.47 (1.24-1.72) | <0.001 | 1.43 (1.17-1.76) | **<0.001** |
| Combined endpoint |  |  |  |  |
| Age, years | 1.08 (1.04-1.12) | <0.001 | 1.03 (0.99-1.07) | 0.112 |
| Male | 0.89 (0.59-1.33) | 0.570 | 1.08 (0.69-1.69) | 0.720 |
| Previous myocardial infarction | 1.36 (0.84-2.19) | 0.201 | 1.10 (0.52-2.31) | 0.795 |
| Atrial fibrillation | 1.93 (1.27-2.94) | 0.002 | 1.05 (0.65-1.70) | 0.819 |
| NYHA functional class ≥III | 1.80 (1.20-2.70) | 0.004 | 1.44 (0.92-2.23) | 0.103 |
| eGFR (per 1 mL/min/1.73 m^2^ increase) | 0.98 (0.97-0.99) | 0.001 | 1.00 (0.98-1.01) | 0.923 |
| Hypertension | 2.39 (1.57-3.64) | <0.001 | 2.29 (1.37-3.83) | **0.002** |
| Diabetes mellitus | 0.97 (0.62-1.53) | 0.927 | 1.00 (0.62-1.63) | 0.978 |
| Aortic valve area (per 1 cm^2^ increase) | 0.55 (0.18-1.65) | 0.291 | 0.37 (0.11-1.23) | 0.107 |
| Surgical or transcatheter AVR (yes/no) | 0.32 (0.21-0.49) | <0.001 | 0.54 (0.32-0.92) | **0.023** |
| Stage of cardiac damage (by each stage increase) | 1.43 (1.25-1.63) | <0.001 | 1.37 (1.17-1.62) | **<0.001** |

**Table S8.** Association of the proposed staging scheme with all-cause mortality and the combined endpoint (mortality and hospitalization for heart failure) in patients with moderate AS.

|  | **Univariable** | | **Multivariable** | |
| --- | --- | --- | --- | --- |
|  | **HR (95% CI)** | ***p* value** | **HR (95% CI)** | ***p* value** |
| All-cause mortality |  |  |  |  |
| Age, years | 1.08 (1.04-1.13) | <0.001 | 1.01 (0.95-1.07) | 0.697 |
| Male | 1.33 (0.74-2.41) | 0.332 | 1.46 (0.77-2.78) | 0.237 |
| Previous myocardial infarction | 2.25 (1.24-4.08) | 0.008 | 1.93 (0.62-6.01) | 0.251 |
| Atrial fibrillation | 3.16 (1.76-5.69) | <0.001 | 2.12 (1.10-4.09) | **0.024** |
| NYHA functional class ≥III | 2.17 (1.12-4.19) | 0.021 | 0.74 (0.36-1.52) | 0.422 |
| eGFR (per 1 mL/min/1.73 m^2^ increase) | 0.96 (0.95-0.98) | <0.001 | 0.97 (0.95-0.99) | **0.018** |
| Hypertension | 1.68 (0.71-3.98) | 0.234 | 1.25 (0.51-3.08) | 0.616 |
| Diabetes mellitus | 0.67 (0.33-1.36) | 0.276 | 0.87 (0.42-1.79) | 0.710 |
| Aortic valve area (per 1 cm^2^ increase) | 0.48 (0.13-1.67) | 0.251 | 0.57 (0.16-2.04) | 0.391 |
| Surgical or transcatheter AVR | 0.40 (0.11-1.44) | 0.405 | 0.61 (0.13-2.81) | 0.533 |
| Stage of cardiac damage (by each stage increase) | 1.44 (1.20-1.73) | <0.001 | 1.15 (0.92-1.43) | 0.216 |
| Combined endpoint |  |  |  |  |
| Age, years | 1.06 (1.02-1.09) | <0.001 | 0.99 (0.94-1.04) | 0.828 |
| Male | 1.14 (0.69-1.87) | 0.602 | 1.00 (0.58-1.71) | 0.993 |
| Previous myocardial infarction | 1.80 (1.06-3.06) | 0.029 | 1.19 (0.66-2.16) | 0.563 |
| Atrial fibrillation | 3.03 (1.82-5.05) | <0.001 | 2.12 (1.20-3.73) | **0.009** |
| NYHA functional class ≥III | 2.58 (1.49-4.46) | <0.001 | 1.46 (0.82-2.61) | 0.195 |
| eGFR (per 1 mL/min/1.73 m^2^ increase) | 0.97 (0.96-0.98) | <0.001 | 0.98 (0.96-0.99) | **0.017** |
| Hypertension | 2.13 (0.97-4.69) | 0.059 | 1.78 (0.78-4.04) | 0.164 |
| Diabetes mellitus | 1.11 (0.64-1.89) | 0.704 | 1.38 (0.79-2.02) | 0.255 |
| Aortic valve area (per 1 cm^2^ increase) | 0.49 (0.16-1.44) | 0.195 | 0.66 (0.22-2.02) | 0.476 |
| Surgical or transcatheter AVR (yes/no) | 0.61 (0.22-1.64) | 0.332 | 0.92 (0.30-2.75) | 0.884 |
| Stage of cardiac damage (by each stage increase) | 1.40 (1.20-1.64) | <0.001 | 1.20 (1.01-1.45) | **0.045** |

**Table S9.** Characteristics of Study Population According to the Proposed Staging Scheme in the validation cohort.

|  | **Stage 0**  **(n = 42)** | **Stage 1**  **(n = 52)** | **Stage 2**  **(n = 40)** | **Stage 3**  **(n = 45)** | **Stage 4**  **(n = 58)** | ***p* value** |
| --- | --- | --- | --- | --- | --- | --- |
| Demographics |  |  |  |  |  |  |
| Age, years | 79 (72-81) | 77 (70-82) | 76 (70-80) | 79 (73-83) | 73 (68-80)†\|\| | **0.038** |
| Male | 17(40) | 31(60) | 30(75) | 21(47) | 31(53) | **0.019** |
| Body surface area (m^2^) | 1.75 (1.62-1.91) | 1.86 (1.69-2.01) | 1.83 (1.68-1.93) | 1.77 (1.64-1.92) | 1.81 (1.71-1.97) | 0.086 |
| Body mass index (kg/m^2^) | 27.1 (26.0-30.2) | 28.4 (25.7-31.2) | 26.8 (23.6-29.6) | 27.4 (24.0-29.7) | 27.4 (24.2-30.9) | 0.334 |
| Cardiovascular risk factors |  |  |  |  |  |  |
| Smoking, n(%) | 9(21) | 6(11) | 3(7) | 3(7) | 9(15) | 0.212 |
| Hypertension, n(%) | 37(88) | 48(92) | 34(85) | 42(93) | 40(69) | **0.002** |
| Diabetes mellitus, n(%) | 10(24) | 16(31) | 9(22) | 12(27) | 13(22) | 0.855 |
| Dyslipidemia, n(%) | 42(100) | 46(88) | 34(85) | 38(84) | 49(84) | 0.117 |
| NYHA functional class ≥III, n(%) | 13(31) | 31(60) | 30(75) | 29(64) | 46(79) | **<0.001** |
| Angina, n(%) | 19(45) | 20(38) | 9(22) | 17(38) | 13(22) | 0.062 |
| Syncope, n(%) | 5(12) | 3(6) | 2(5) | 4(9) | 2(3) | 0.497 |
| Coronary artery disease, n(%) | 14(33) | 22(42) | 20(50) | 27(60) | 24(41) | 0.123 |
| Estimated glomerular filtration rate (mL/min/1.73 m^2^) | 61.9 (44.8-84.3) | 64.1 (54.3-84.0) | 64.8 (44.5-79.6) | 53.5 (36.9-70.4) | 56.5 (48.1-78.2) | 0.206 |
| Systolic blood pressure (mmHg) | 130 (118-140) | 120 (110-140) | 115 (100-126)† | 120 (110-130)† | 115 (98-130)†‡\|\| | **0.001** |
| Diastolic blood pressure (mmHg) | 70 (60-80) | 70 (60-80) | 60 (60-76) | 70 (60-70) | 70 (60-80) | 0.275 |
| Heart rhythm at the time of TTE |  |  |  |  |  | <0.001 |
| Sinus rhythm | 42 (100) | 46(89) | 27(68) | 34(76) | 31(53) |  |
| Atrial fibrillation | 0 (0) | 6(11) | 13(32) | 11(24) | 27(47) |  |
| Aortic stenosis |  |  |  |  |  | 0.257 |
| Moderate | 4(9) | 6(11) | 2(5) | 1(2) | 2(3) |  |
| Severe | 38(91) | 46(89) | 38(95) | 44(98) | 56(97) |  |
| TTE characteristics |  |  |  |  |  |  |
| Relative wall thickness | 0.58 (0.51-0.69) | 0.64 (0.50-0.78) | 0.65 (0.48-0.74) | 0.65 (0.51-0.80) | 0.55 (0.44-0.65)‡§\|\| | **0.010** |
| Septal wall thickness (cm) | 1.3 (1.2-1.6) | 1.5 (1.4-1.7)† | 1.5 (1.3-1.9)† | 1.6 (1.3-1.8)† | 1.5 (1.3-1.6)‡ | **0.007** |
| Posterior wall thickness (cm) | 1.2 (1.1-1.5) | 1.5 (1.3-1.7)† | 1.4 (1.3-1.8)† | 1.5 (1.3-1.7)† | 1.4 (1.2-1.5)‡§\|\| | **<0.001** |
| LVED diameter (cm) | 4.2 (3.9-4.7) | 4.6 (4.0-5.0)† | 4.9 (4.2-5.4)† | 4.5 (4.1-5.0)† | 5.0 (4.4-5.7)†‡\|\| | **<0.001** |
| LV mass (g/m^2^) | 120 (104-140) | 159 (133-180)† | 175 (135-220)† | 165 (122-206)† | 160 (134-198)† | **<0.001** |
| LVEDV (mL/m^2^) | 33 (27-39) | 46 (36-54)† | 55 (37-69)†‡ | 44 (35-53)†§ | 46 (35-63)† | **<0.001** |
| LVESV (mL/m^2^) | 20 (14-27) | 25 (19-31) | 34 (24-43)†‡ | 25 (21-31)†§ | 34 (26-46)†‡\|\| | **<0.001** |
| LVSV (mL/m^2^) | 50 (45-60) | 47 (41-59) | 39 (33-50)†‡ | 43 (38-57)† | 36 (29-49)†‡\|\| | **<0.001** |
| LVEF (%) | 55.0 (51.0-58.8) | 53.0 (48.5-60.1) | 47.0 (40.2-53.3)†‡ | 52.1 (44.5-56.2)§ | 44.9 (34.5-54.5)†‡\|\| | **<0.001** |
| LAV (mL/m^2^) | 27 (20-40) | 38 (21-49)† | 45 (22-61)† | 47 (21-58)† | 47 (22-57) | **0.011** |
| MR ≥ moderate | 4(9) | 12(23) | 7(17) | 8(18) | 28(48) | **<0.001** |
| PASP (mmHg) | 30 (27-33) | 30 (23-36) | 35 (29-44)†‡ | 50 (38-63)†‡§ | 52 (39-62)†‡§ | **<0.001** |
| TR ≥ moderate | 0(0) | 0(0) | 0(0) | 38(84) | 41(71) | **<0.001** |
| Mean aortic valve gradient (mmHg) | 48.9 (40.7-62.3) | 55.0 (44.6-69.2) | 49.6 (41.2-59.9) | 55.6 (45.3-68.9) | 49.2 (42.7-63.0) | 0.452 |
| Aortic valve area (cm^2^) | 0.8 (0.68-1.01) | 0.81 (0.67-0.99) | 0.72 (0.53-0.85)‡ | 0.72 (0.55-0.87)†‡ | 0.63 (0.47-0.83)†‡ | **<0.001** |
| LV global LS, % | 18.2 (16.2-19.9) | 13.3 (11.3-15.0)† | 9.9 (6.9-12.8)‡ | 14.1 (11.3-16.6)†§ | 8.7 (6.0-12.8)†‡\|\| | **<0.001** |
| RV free-wall LS, % | 25.0 (21.7-29.0) | 22.5 (18.1-25.8)† | 22.0 (17.9-24.9)† | 25.2 (21.7-27.3)‡§ | 14.2 (10.4-16.6)†‡§ \|\| | **<0.001** |
| Peak atrial LS, % | 20.0 (16.4-24.7) | 15.2 (13.2-18.8)† | 8.1 (6.4-10.4)†‡ | 11.1 (8.2-18.1)†‡§ | 6.5 (4.4-8.6)†‡§\|\| | **<0.001** |

Values are mean (95% CI) or n(%). *The p values depict differences between stages of extra-aortic valvular cardiac abnormalities: †p < 0.05 vs. Group 0 with Bonferroni’s post hoc analysis. ‡p < 0.05 vs. Group 1 with Bonferroni’s post hoc analysis. §p < 0.05 vs. Group 2 with Bonferroni’s post hoc analysis. ||p < 0.05 vs. Group 3 with Bonferroni’s post hoc analysis.

Abbreviations: COPD, chronic obstructive pulmonary disease; GFR, glomerular filtration rate; GLS, global longitudinal strain; LV, left ventricle/ventricular, LS, longitudinal strain; MR, mitral regurgitation; PASP, pulmonary artery systolic pressure; RV right ventricle/ventricular; TR, tricuspid regurgitation.

**Table S10.** Association of the proposed staging scheme with all-cause mortality and the combined endpoint (mortality and hospitalization for heart failure) in the validation cohort.

|  | **Univariable** | | **Multivariable** | |
| --- | --- | --- | --- | --- |
|  | **HR (95% CI)** | ***p* value** | **HR (95% CI)** | ***p* value** |
| All-cause mortality |  |  |  |  |
| Age, years | 1.02 (0.99-1.05) | 0.172 | 1.02 (0.987-1.07) | 0.181 |
| Male | 1.53 (0.96-2.45) | 0.072 | 1.99 (1.18-3.34) | **0.009** |
| Previous myocardial infarction | 1.35 (0.81-2.01) | 0.201 | 1.10 (0.52-1.99) | 0.559 |
| Atrial fibrillation | 2.27 (1.42-3.70) | <0.001 | 1.64 (0.93-2.85) | 0.086 |
| NYHA functional class ≥III | 1.47 (0.90-2.41) | 0.123 | 1.21 (0.69-2.13) | 0.495 |
| eGFR (per 1 mL/min/1.73 m^2^ increase) | 0.99 (0.98-1.00) | 0.088 | 0.992 (0.97-1.00) | 0.205 |
| Hypertension | 0.61 (0.34-1.08) | 0.095 | 0.61 (0.33-1.12) | 0.114 |
| Diabetes mellitus | 1.03 (0.61-1.74) | 0.895 | 1.34 (0.77-2.33) | 0.296 |
| Aortic valve area (per 1 cm^2^ increase) | 0.63 (0.27-1.48) | 0.291 | 0.67 (0.22-2.04) | 0.492 |
| Surgical or transcatheter AVR | 0.38 (0.19-0.77) | 0.008 | 0.28 (0.12-0.62) | **0.002** |
| Severe aortic stenosis | 1.42 (0.52-3.91) | 0.489 | 1.10 (0.32-3.72) | 0.870 |
| Stage of cardiac damage (by each stage increase) | 1.35 (1.15-1.59) | <0.001 | 1.24 (1.01-1.51) | **0.033** |
| Combined endpoint |  |  |  |  |
| Age, years | 1.00 (0.97-1.03) | 0.757 | 1.00 (0.97-1.03) | 0.717 |
| Male | 1.52 (1.01-2.28) | 0.042 | 2.11 (1.33-3.34) | **0.001** |
| Previous myocardial infarction | 1.36 (0.84-2.19) | 0.321 | 1.10 (0.52-2.31) | 0.654 |
| Atrial fibrillation | 2.27 (1.49-3.70) | <0.001 | 1.41 (0.86-2.27) | 0.171 |
| NYHA functional class ≥III | 1.47 (0.96-2.26) | 0.073 | 1.02 (0.63-1.67) | 0.919 |
| eGFR (per 1 mL/min/1.73 m^2^ increase) | 0.99 (0.98-1.00) | 0.070 | 0.99 (0.98-1.00) | 0.101 |
| Hypertension | 0.55 (0.34-0.91) | 0.021 | 0.68 (0.40-1.16) | 0.162 |
| Diabetes mellitus | 0.74 (0.46-1.19) | 0.221 | 0.750 (0.45-1.23) | 0.255 |
| Aortic valve area (per 1 cm^2^ increase) | 0.45 (0.21-0.94) | 0.036 | 0.42 (0.16-1.12) | 0.086 |
| Surgical or transcatheter AVR (yes/no) | 0.42 (0.12-0.81) | 0.010 | 0.37 (0.18-0.76) | **0.007** |
| Severe aortic stenosis | 1.13 (0.52-2.44) | 0.754 | 0.47 (0.18-1.22) | 0.123 |
| Stage of cardiac damage (by each stage increase) | 1.50 (1.30-1.74) | <0.001 | 1.44 (1.20-1.72) | **<0.001** |

**Table S11.** Net reclassification improvement (NRI) and integrated discrimination improvement (IDI) analysis compared to previous classifications of extravalvular cardiac damage at 2-year follow-up in the validation cohort.

| **Primary endpoint (all-cause death)** | | | | |
| --- | --- | --- | --- | --- |
|  | **Index** | **Estimate** | **95% CI** | ***p* value** |
| Proposed staging vs. Généreux et al.[5] | NRI | 0.307 | 0.006 – 0.460 | 0.04 |
| Proposed staging vs. Généreux et al.[5] | IDI | 0.023 | 0.001 – 0.046 | 0.03 |
| Proposed staging vs. Tastet et al.[10] | NRI | 0.361 | -0.005 – 0.507 | 0.08 |
| Proposed staging vs. Tastet et al.[10] | IDI | 0.026 | 0.002 – 0.057 | 0.02 |
| **Secondary endpoint (all-cause death and hospitalization for HF)** | | | | |
|  | **Index** | **Estimate** | **95% CI** | ***p* value** |
| Proposed staging vs. Généreux et al.[5] | NRI | 0.363 | 0.067 – 0.454 | <0.001 |
| Proposed staging vs. Généreux et al.[5] | IDI | 0.031 | 0.007 – 0.060 | 0.01 |
| Proposed staging vs. Tastet et al.[10] | NRI | 0.420 | 0.035 – 0.559 | 0.03 |
| Proposed staging vs. Tastet et al.[10] | IDI | 0.043 | 0.013 – 0.082 | 0.01 |

**Table S12.** Intra- and inter-observer reproducibility of longitudinal strain measurements.

| **Variable** | **ICC (95% CI)** | **CV (%)** |
| --- | --- | --- |
| **LV GLS (intraobserver)** | **0.96 (0.93-0.99)** | **4.1** |
| **LV GLS (interobserver)** | **0.95 (0.86-0.98)** | **8.6** |
| **PALS (intraobserver)** | **0.98 (0.97-0.99)** | **12.2** |
| **PALS (interobserver)** | **0.96 (0.91-0.98)** | **30.1** |
| **RV FWS (intraobserver)** | **0.98 (0.97-0.99)** | **4.9** |
| **RV FWS (interobserver)** | **0.96 (0.91-0.98)** | **7.0** |

*Abbreviations: CI, confidence interval; CV, coefficient of variation; ICC, intraclass correlation coefficient; LV GLS, left ventricular global longitudinal strain; PALS, peak atrial longitudinal strain; RV FWLS, right ventricular free wall longitudinal strain.*
